# Phytoplasma Effector SAP54 Hijacks Plant Reproduction by Degrading MADS-box Proteins and Promotes Insect Colonization in a RAD23-Dependent Manner

**DOI:** 10.1371/journal.pbio.1001835

**Published:** 2014-04-08

**Authors:** Allyson M. MacLean, Zigmunds Orlovskis, Krissana Kowitwanich, Anna M. Zdziarska, Gerco C. Angenent, Richard G. H. Immink, Saskia A. Hogenhout

**Affiliations:** 1 Department of Cell and Developmental Biology, John Innes Centre, Norwich Research Park, Norwich, Norfolk, United Kingdom; 2 Bioscience, Plant Research International, Wageningen, The Netherlands; 3 Laboratory of Molecular Biology, Wageningen University, Wageningen, The Netherlands; University of Pennsylvania, United States of America

## Abstract

Pathogens that rely upon multiple hosts to complete their life cycles often modify behavior and development of these hosts to coerce them into improving pathogen fitness. However, few studies describe mechanisms underlying host coercion. In this study, we elucidate the mechanism by which an insect-transmitted pathogen of plants alters floral development to convert flowers into vegetative tissues. We find that phytoplasma produce a novel effector protein (SAP54) that interacts with members of the MADS-domain transcription factor (MTF) family, including key regulators SEPALLATA3 and APETALA1, that occupy central positions in the regulation of floral development. SAP54 mediates degradation of MTFs by interacting with proteins of the RADIATION SENSITIVE23 (RAD23) family, eukaryotic proteins that shuttle substrates to the proteasome. Arabidopsis *rad23* mutants do not show conversion of flowers into leaf-like tissues in the presence of SAP54 and during phytoplasma infection, emphasizing the importance of RAD23 to the activity of SAP54. Remarkably, plants with SAP54-induced leaf-like flowers are more attractive for colonization by phytoplasma leafhopper vectors and this colonization preference is dependent on RAD23. An effector that targets and suppresses flowering while simultaneously promoting insect herbivore colonization is unprecedented. Moreover, RAD23 proteins have, to our knowledge, no known roles in flower development, nor plant defence mechanisms against insects. Thus SAP54 generates a short circuit between two key pathways of the host to alter development, resulting in sterile plants, and promotes attractiveness of these plants to leafhopper vectors helping the obligate phytoplasmas reproduce and propagate (zombie plants).

## Abstract 2

The phytoplasma bacterial plant parasite depends on leafhopper insects to spread and propagate itself. This study reveals how phytoplasma subverts plant development to turn flowers into leaves and thus make its host more attractive to leafhoppers.

## Author Summary

Parasites that colonize multiple hosts often coerce these hosts into improving their own survival and reproduction rates. However, how parasites do this is largely unknown. Phytoplasmas are bacterial plant parasites that require sap-feeding insect vectors—leafhoppers—for their propagation and dispersal. It has been known for a long time that phytoplasmas stimulate dramatic developmental changes in a broad range of plant species, such as the conversion of flowers into leaves known as phyllody and the proliferation of stems known as “witches' broom.” Here we report how and why phytoplasmas cause these dramatic developmental changes. We identified a phytoplasma virulence protein, SAP54, which transforms flowers into leaves and converts plants into more attractive hosts for the egg-laying and reproduction of their leafhopper vectors. We show that SAP54 exerts its effect by promoting the degradation of proteins that regulate important developmental processes in flowering plants. These proteins are highly conserved transcription factors of the MADS-box family, and reducing their activity through SAP54–mediated degradation curtails flower development, generating sterile plants. This degradation process requires RAD23, a protein that recruits the transcription factors to the protein degradation machinery. The resulting sterile plants, which form leaves in place of flowers, are more attractive to leafhoppers, arguably making phytoplasmas master manipulators of the parasite world.

## Introduction

Microorganisms that inhabit eukaryotic hosts must adapt themselves to these living environments, or manipulate their hosts to permit colonization. A classical example of the latter is the release of proteineaceous effectors (i.e., virulence proteins) by microbial pathogens to modulate processes of their eukaryotic hosts, with most effectors acting either directly or indirectly to suppress host defence responses. Prominent examples of these include type III effectors of Gram-negative bacteria such as *Pseudomonas* and *Salmonella* [1], RXLR and Crinkler proteins secreted by oomycetes [2], and the TAL effectors of *Xanthomonas*, which bind to host promoters and misregulate gene expression [3]. By these means, the pathogen impairs the host's ability to defend itself, thereby promoting host susceptibility to the invading microorganism.

Pathogens and parasites may also manipulate the behavior and development of their hosts. The protozoan *Toxoplasma gondii* modifies the behaviour of rats in response to the scent of cat urine, reprogramming the rat's behavioural responses to increase its likelihood of predation [4]. Similarly, the lancet liver fluke infects the brain of ants and compels the insect to climb to the top of a blade of grass and remain motionless until ingested by a grazing ruminant. Thus, these parasites coerce host behavior to improve their opportunities for transmission to a new host. Parasites may also influence host development, with known examples including a pathogenic fungus (*Puccinia monoica*) that stimulates the growth of pseudo-flowers from infected plant hosts to attract insects that subsequently “pollinate” the fungus [5] and bacterial pathogens that alter the profile of organic volatiles released from infected plants as a means of attracting insect vectors [6]. However, molecular mechanisms by which pathogens alter either host behaviour or development are largely unknown.

Host coercion is particularly important for obligate pathogens that are completely dependent on their hosts. Phytoplasma are bacterial plant pathogens that have a dual host life cycle that is dependent on sap-feeding insects for transmission to plants [7]. Insect vectors (planthoppers, leafhoppers, and psyllids) acquire phytoplasma by ingesting the phloem of infected plants. The insect vectors become competent to transmit the bacteria to healthy plants following the colonization of salivary glands by phytoplasma, which are subsequently released into the phloem with saliva during insect feeding. Aster Yellows phytoplasma strain Witches' Broom (AY-WB) can infect a broad range of plants, eliciting symptoms such as phyllody (conversion of flowers into leaf-like structures), virescence (greening of floral organs such as petals and stamens), and witches' brooms (increased proliferation of stems) reflecting perturbations in host development that are beneficial to AY-WB or its insect vector [7],[8]. We have previously identified an AY-WB effector protein (called SAP54) that transforms flowers into leaf-like vegetative tissues when expressed in *Arabidopsis thaliana* (hereafter Arabidopsis) [9]. Healthy Arabidopsis flowers are determinate structures with APETALA1 (AP1) regulating gene expression programs during the establishment of floral meristems [10]. Flowers of AY-WB–infected plants and SAP54-expressing transgenic Arabidopsis lines exhibit a loss of floral determinacy, reflected by vegetative shoots arising from the center of the flower and from the axil of the first whorl organs. We herein reveal the mechanism by which AY-WB phytoplasma coerces the plant host into suppressing its floral development to the benefit of this pathogen and its insect vector, but at the expense of the plant host reproductive success.

## Results

### Phytoplasma Effector SAP54 Interacts with MADS-Domain Transcription Factors

**Figure 1:**
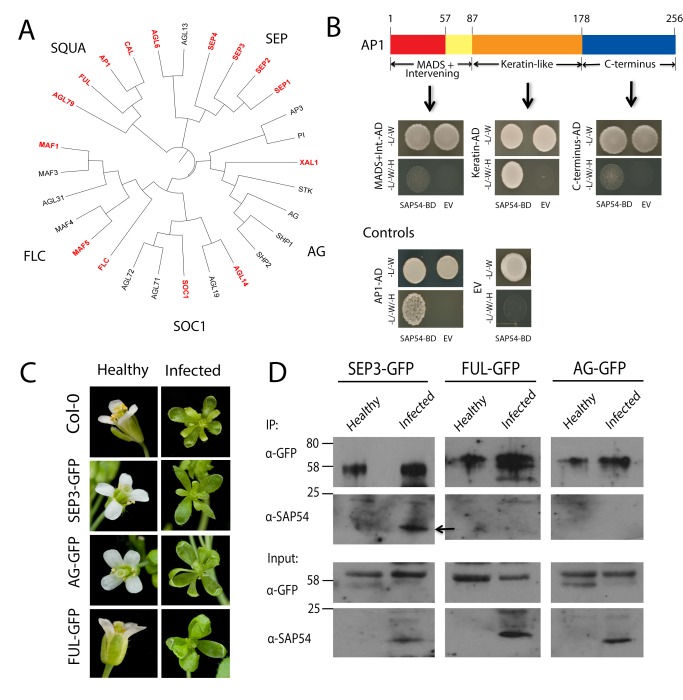
Phytoplasma SAP54 interacts specifically with the Keratin-like (K) domain of selected Type II MADS-box transcription factors (MTFs). (A) A comprehensive yeast two-hybrid screen of 106 Arabidopsis MTFs reveals that SAP54 interacts with members of the Type II subfamily of MTFs (proteins that interact with SAP54 are indicated in red font). For simplicity, not all MTFs are included in the phylogenetic tree. (B) SAP54 interacts primarily with the K domain of AP1. AD, GAL4-activation domain; BD, GAL4-DNA binding domain; EV, empty vector control. (C) Flowers produced from healthy (left) and AY-WB–infected (right) Arabidopsis lines approximately 4 wk postinoculation. (D) SAP54 (indicated by an arrow) co-immunoprecipitates with SEP3–GFP but not FUL–GFP or AG–GFP. Flowers for immunoprecipitation experiments were harvested from transgenic lines pictured in panel C at an early point of infection (approximately 2 wk postinoculation) to minimize MTF loss due to destabilization. Equal loading of samples was confirmed via Bradford assays to quantify protein concentration.

We wished to investigate how AY-WB phytoplasma alters flower architecture in infected plants. A yeast two-hybrid screen against an Arabidopsis seedling library (with SAP54 as bait) identified the Type II MADS-domain transcription factors (MTFs) AGAMOUS-LIKE 12 (AGL12), MADS AFFECTING FLOWERING1 (MAF1), and SEPALLATA3 (SEP3) as SAP54 interactors (Table S1). To investigate the breadth of interactions between SAP54 and MTFs, we examined SAP54 interaction with 106 Arabidopsis MTFs in a matrix-based yeast two-hybrid screen [11]. This confirmed SAP54 interaction with AGL12, MAF1, and SEP3, and furthermore identified 12 additional interacting partners (Figure 1A and Table S2), including the well-characterized floral meristem identity and homeotic proteins AP1 [10], and SEP3 paralogues SEP1, SEP2, and SEP4. SAP54 interacts solely with Type II MTFs and not with Type I MTFs in the two-hybrid experiments, indicating that SAP54 primarily targets the MTFs involved in floral transition and floral organ development.

Type II MTFs are modular proteins consisting of four domains. We found that SAP54 interacts primarily with the Keratin-like (K) domain of AP1 (Figure 1B) and not with the more highly conserved MADS domain that is present in both plant and animal MTFs. The K domain contributes to the formation of MTF protein–protein interactions when these regulators associate as dimers and quartets [12],[13], and a classical K domain is specific to plant Type II MTFs [14]. Therefore SAP54 may have evolved to selectively target plant MTFs (that contain a K domain) and not those of insects (that lack this domain), an important characteristic given that phytoplasmas effectively colonize many organs of their insect vectors [7].

To assess whether SAP54 interacts with MTFs in AY-WB–infected plants, we made use of Arabidopsis transgenic lines that express translational fusions of SEP3–GFP, FUL–GFP, or AG–GFP under control of their native promoters [15] to conduct co-immunoprecipitation experiments. The transgenic lines produce leaf-like flowers that are indistinguishable from those of wild-type Arabidopsis plants when infected with phytoplasma (Figure 1C), indicating that these lines are suitable to the study of SAP54–MTF interaction. To minimize MTF loss due to SAP54 destabilization (described below), we harvested flowers of transgenic lines shortly after infection with AY-WB (2 wk postinoculation) at a stage when flowers exhibit a normal (non-leaf-like) appearance, yet SAP54 is present in infected plants. SAP54 co-immunoprecipitated with SEP3–GFP in samples of AY-WB–infected plants, but no corresponding protein was detected in healthy controls (Figure 1D and Table S3). In contrast, SAP54 did not co-immunoprecipate with FUL–GFP or AG–GFP (Figure 1D). This experiment confirms that AY-WB produces SAP54 during infection of Arabidopsis and that SAP54 interacts with SEP3 in AY-WB–infected plants. As well, while SAP54 interacts with 15 MTFs in a yeast two-hybrid system (Figure 1A), it is possible that only a subset of these interactions occur *in planta*. To address this, we conducted an immunoprecipitation experiment of GFP–SAP54 from *35S:GFP*–*SAP54* transgenic Arabidopsis followed by a mass spectrometry analysis to identify interacting proteins. Peptides associated with Type II MTFs MAF1, SUPPRESSOR OF OVEREXPRESSION OF CONSTANS1 (SOC1), SEP1, SEP2, and AP1 were recovered in samples immunoprecipitated with GFP–SAP54 but not with GFP alone (Table S4). Thus, we conclude that SAP54 indeed interacts with MTFs in infected plants, and that many of the MTFs identified as SAP54 interactors in the yeast two-hybrid screens also interact with SAP54 *in planta*.

### SAP54 Destabilizes MTFs in an Ubiquitin/26S Proteasome-Dependent Manner

**Figure 2:**
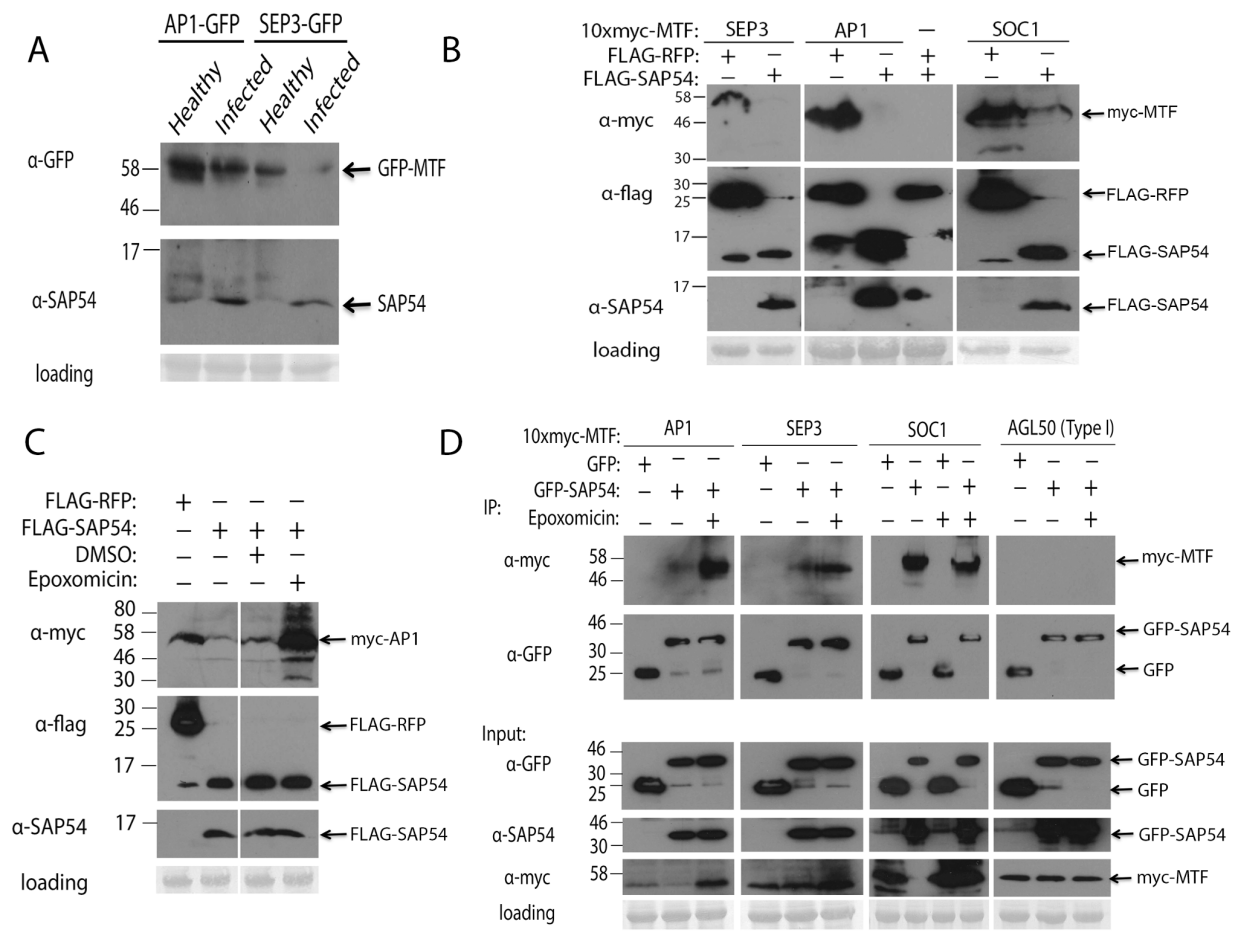
Phytoplasma SAP54 interacts with and destabilizes MADS-box transcription factors in plants. (A) MTFs AP1 and SEP3 are destabilized in AY-WB–infected Arabidopsis lines. Flowers from healthy and phytoplasma-infected plants were harvested approximately 4 wk postinoculation. (B) MTFs are destabilized when expressed in the presence of SAP54. 10xmyc-tagged MTFs were transiently co-expressed with flag-tagged SAP54 or an RFP control in agroinfiltrated *N. benthamiana* leaves. (C) SAP54-mediated destabilization of AP1 is inhibited by epoxomicin. Infiltrated tissues were treated with 50 µM epoxomicin (dissolved in DMSO) 8 h prior to harvest. (D) MTFs AP1, SEP3, and SOC1 co-immunoprecipitate with GFP-tagged SAP54. Co-immunoprecipitation experiments of these Type II MTFs were performed alongside Type I MTF AGL50, which was not detected. Proteins were transiently expressed in *N. benthamiana* in the presence or absence of 50 µM epoxomicin to stabilize MTFs.

The floral architecture of SAP54-expressing and AY-WB–infected Arabidopsis resembles that of higher order *sep* mutants (i.e., loss of floral determinacy and conversion of floral organs into leaf-like structures [16],[17]), and we hypothesized that SAP54 may act to perturb MTF function. Western blots performed using flowers collected from healthy and AY-WB–infected Arabidopsis lines expressing AP1–GFP or SEP3–GFP revealed that these MTFs appear to be less abundant in infected leaf-like flowers harvested at a late stage of infection (4+ wk postinoculation) (Figure 2A and Table S5), suggesting that phytoplasma may act to destabilize these transcription factors. Phytoplasma are obligate biotrophs that are genetically intractable, and thus we were unable to generate an AY-WB *SAP54* mutant. Therefore, we examined the interaction between SAP54–MTFs more closely by co-expression assays in *Nicotiana benthamiana* using pTRBO-based vectors, which are *Tobacco mosaic virus*–based expression vectors that allow for higher levels of *in planta* protein production compared with 35S constructs [8],[18]. We observed that whereas Type II MTFs were detected on immunoblots when transiently co-expressed with a control protein (pTRBO::*Flag-RFP*), the accumulation of AP1, SEP3, and SOC1 was much reduced or undetectable when co-expressed as 10xmyc-tagged proteins with pTRBO::*Flag-SAP54* (Figure 2B and Table S6). In contrast, the accumulations of non-SAP54 interacting Type I MTFs AGL50, AGL62, and AGL80 in the presence of SAP54 were not or only weakly reduced (Figure S1A). SAP54 interacted with members of the Type II canonical MADS-box proteins (MIKC^C^) in yeast two-hybrid screens, but not with MIKC* proteins, which have a distinct Keratin-like domain [19]. Consistent with this is the observation that the Type II MIKC* type AGL66 was not destabilized in the presence of SAP54 in *N. benthamiana* (Figure S1B). Thus, the phytoplasma effector SAP54 appears to selectively destabilize Type II MIKC^C^ MTFs that are the key regulators of floral organ formation in flowering plants.

SAP54 may act directly as a protease to catalyze the proteolysis of select MTFs, or alternatively, this effector may exploit a host mechanism, such as the ubiquitin/26S proteasome system (UPS) to degrade MTFs. Treatment of infiltrated samples with a protease inhibitor cocktail did not affect SAP54 activity (Figure S1C), however treatment with epoxomicin, a potent inhibitor of the UPS [20], prevented the SAP54-mediated destabilization of AP1 (Figure 2C, Table S7, and Figure S1C). Thus, SAP54 is likely to degrade MTFs via the host UPS. All eukaryotes have a UPS, including yeast. Nonetheless, SAP54 did not degrade the MTFs in yeast two-hybrid experiments in which SAP54 was fused to the GAL4 DNA binding domain (GAL4BD–SAP54) and Type II MIKC^C^ MTFs to the GAL4 activation domain (GAL4AD–AP1, GAL4AD–SEP3, and GAL4AD–SOC1) (Figure S1D). Therefore, the SAP54-mediated degradation of MTFs may require a helper protein(s) from the plant host.

We lastly employed the transient expression system in *N. benthamiana* to confirm the interactions between SAP54 and MTFs *in planta*. 10xmyc-tagged AP1, SEP3, and SOC1 readily co-immunoprecipitate with GFP-tagged SAP54 (but not GFP), whereas Type I AGL50 (Type I MTF) was not pulled down with SAP54 (Figure 2D, Tables S8, S9).

### SAP54 Interacts with Ubiquitin Binding Proteins RAD23C and RAD23D

**Figure 3:**
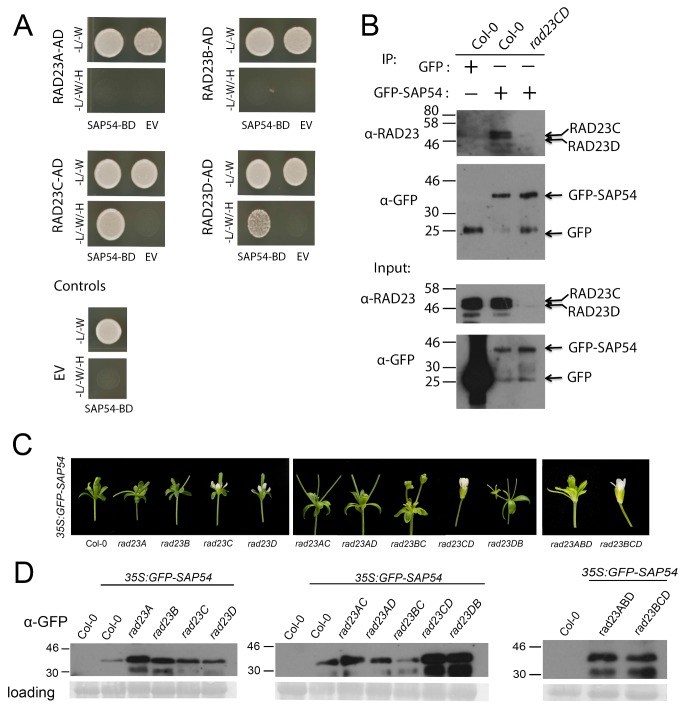
Phytoplasma SAP54 interacts with Arabidopsis RAD23 proteins. (A) SAP54 interacts with Arabidopsis RAD23C and RAD23D but not RAD23A or RAD23B isoforms in yeast two-hybrid assays. (B) RAD23C (44 kDa) and RAD23D (40 kDa) co-immunoprecipitate with GFP–SAP54 in samples obtained from transgenic Arabidopsis expressing *35S:GFP–SAP54*. We did not detect RAD23 following immunoprecipitation of GFP (in transgenic Arabidopsis expressing *35S:GFP*), nor did we detect an interaction with RAD23A or RAD23B in an Arabidopsis *rad23CD* double mutant. Equal loading of samples was verified via Bradford assays to confirm protein concentration. (C) Flowers produced from transgenic lines expressing *35S:GFP–SAP54* in wild-type (Col-0) and *rad23* mutant Arabidopsis lines indicate that SAP54-induced phyllody requires RAD23C and RAD23D. (D) Western blot analysis reveals GFP–SAP54 expression levels in rosette leaves harvested from plants in panel C. GFP–SAP54 is indicated by an arrow. AD, GAL4-activation domain; BD, GAL4–DNA binding domain; EV, empty vector control.

In addition to the MTFs, the yeast two-hybrid screen against the Arabidopsis seedling library revealed that SAP54 interacts with RADIATION SENSITIVE23 (RAD23) family isoforms RAD23C and RAD23D (Table S1). RAD23 proteins have been proposed to act as shuttle proteins to deliver ubiquitinated substrates to the UPS for degradation in eukaryotes [21]. Arabidopsis encodes four RAD23 isoforms, however the yeast two-hybrid data indicate that SAP54 interacts specifically with RAD23C and RAD23D but not RAD23A or RAD23B (Figure 3A). This is consistent with the observation that RAD23C and RAD23D co-immunoprecipitate in the presence of GFP–SAP54 (but not GFP alone) (Figure 3B) in Arabidopsis. RAD23 proteins did not co-immunoprecipitate with GFP–SAP54 in the *rad23CD* mutant (Figure 3B), indicating that SAP54 prefers to interact with RAD23C and RAD23D as opposed to RAD23A and RAD23B *in planta*.

### RAD23 Proteins Are Essential for Phytoplasma-Induced Phyllody

With the consideration that SAP54 degrades MTFs and that RAD23 proteins act to shuttle poly-ubiquitinated substrates to the UPS, we hypothesized that the Arabidopsis RAD23 proteins may be required for SAP54-mediated degradation. Although the RAD23 proteins are essential, the proteins have largely redundant functions and *rad23* single mutants and the majority of *rad23* double mutants do not exhibit any obvious developmental defects (Figure S2), although higher order *rad23B* mutants do demonstrate various pleiotropic phenotypes (i.e., shorter stature, smaller siliques, and reduced seed production) [21].

To investigate if RAD23 proteins contribute to SAP54-induced leaf-like flowers, homozygous Arabidopsis *rad23* T-DNA mutant lines [21] were transformed with *35S:GFP–SAP54* (Figure 3C). In a wild-type Arabidopsis background, expression of *GFP–SAP54* induces a strong degree of phyllody (growth of leaf-like flowers), virescence (greening of floral organs), and a frequent loss of floral determinacy as evidenced by the outgrowth of stems from the centre of the flower. However, approximately one half of *35S:GFP–SAP54–*expressing transformants obtained from *rad23C* (48 of 109 transgenic plants) and *rad23D* (8 of 22 transgenic plants) mutant lines exhibited a milder degree of phyllody, with loss of determinacy typically restricted to the early onset flowers (Table 1 and Figure S3). Moreover, the majority of transformants originating from *rad23CD* double mutants (124 of 138 transgenic plants) and *rad23BCD* triple mutants (50 of 64 transgenic plants) produced flowers that displayed no signs of phyllody or virescence (Figure 3C). Western blot analysis confirmed the expression of the GFP-tagged SAP54 in the transgenic lines (Figure 3D) and revealed that the mild phyllody observed in a minority of *rad23CD* transgenic lines (14 of 138 transgenic plants) was likely due to a very high level of SAP54 expression (Figure S4). In contrast, *rad23AC*, *rad23AD*, *rad23BC*, *rad23BD*, and *rad23ABD* mutant transgenic lines produced flowers comparable to those observed in a wild-type (Col-0) background (Figure 3C, Table 1). Thus, the SAP54-mediated degradation of MTFs is dependent predominantly on RAD23C and RAD23D, whereas other RAD23 isoforms may be involved depending on SAP54 abundance.

**Table 1:** Phenotype scoring of *35S:GFP–SAP54* transgenic lines.

Degree of Phyllody and Virescence				
Genotype	Absent	Mild	Strong	Total No. Plants
Col-0	0	8	26	34
*rad23A*	0	10	69	79
*rad23B*	0	14	68	82
*rad23C*	0	48	61	109
*rad23D*	0	8	14	22
*rad23AC*	0	9	35	44
*rad23AD*	0	6	9	15
*rad23BC*	0	2	23	25
*rad23BD*	0	8	12	20
*rad23CD*	124	14	0	138
*rad23ABD*	0	4	49	53
*rad23BCD*	50	14	0	64

**Figure 4:**
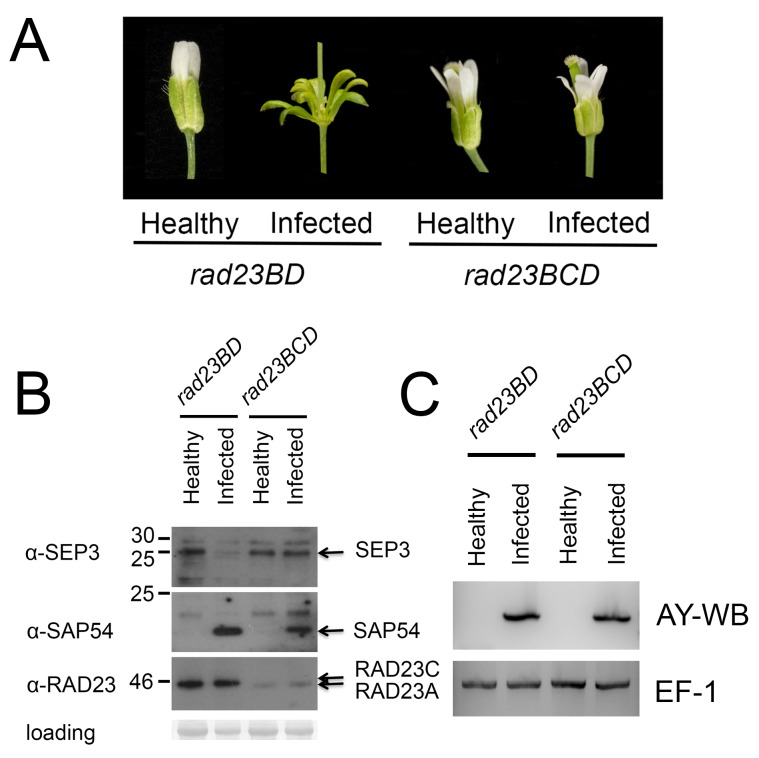
Arabidopsis *rad23BCD* triple mutants do not exhibit symptoms of virescence or phyllody when infected with AY-WB. (A) Flowers produced from AY-WB–infected *rad23BD* mutants produce leaf-like flowers, whereas infected *rad23BCD* mutants grow flowers that resemble those of healthy plants. (B) Western blot analysis reveals that SEP3 is destabilized in *rad23BD* leaf-like flowers but not in *rad23BCD* flowers. SAP54 was detected in flowers harvested from AY-WB–infected *rad23* mutants but not healthy Arabidopsis plants. (C) The infection status of plants in panel A was confirmed using primers specific for AY-WB.

To assess whether the Arabidopsis RAD23 proteins are also required for SAP54-mediated MTF destabilization during AY-WB infection, the various *rad23* single, double, and triple T-DNA insertion mutants were infected with AY-WB phytoplasma. AY-WB–infected *rad23BCD* triple mutants produced determinate flower-like organs that resemble those of healthy wild-type Col-0 plants (Figure 4). The degradation of MTF SEP3 was lost in the phytoplasma-infected *rad23BCD* mutant, whereas degradation of this MTF was observed in *rad23BD* mutant (with leaf-like flowers) (Figure 4). Remarkably these plants still showed other symptoms of infection (Figure S5), such as the witches' brooms that are typically observed in AY-WB–infected Arabidopsis plants [7]. In contrast, the *rad23* single and double mutants, including *rad23BD* and *rad23CD* mutant lines, and the *rad23ABD* triple mutants produced leaf-like indeterminate flowers that resemble those of AY-WB–infected wild-type Col-0 plants (Figure 4, Figure S6). We conclude from these results that the SAP54-mediated degradation of MTFs requires predominantly RAD23C and RAD23D, but that RAD23B may also facilitate this process in infected hosts.

### SAP54 Enhances Phytoplasma Insect Vector Colonization in a RAD23-Dependent Manner

**Figure 5:**
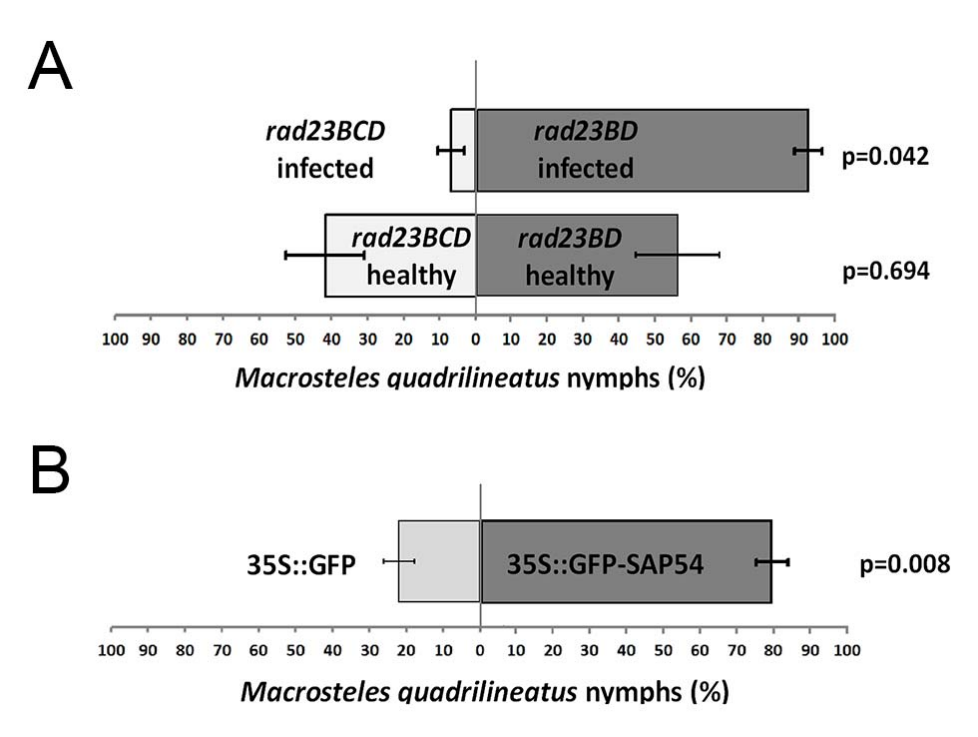
Aster leafhopper *Macrosteles quadrilineatus* demonstrates oviposition preference for plants with leaf-like flowers. (A) *M. quadrilineatus* produces significantly more progeny on AY-WB–infected *rad23BD* mutants (leaf-like flower phenotype) compared to *rad23BCD* mutant plants (non-leaf-like flower phenotype) (*t*_(5)_ = 4.7; *p* = 0.042). Insects do not exhibit a preference between healthy *rad23BD* and *rad23BCD* plants (*t*_(5)_ = 0.45; *p* = 0.694). (B) *M. quadrilineatus* adults produce more nymphs on transgenic Arabidopsis expressing GFP-tagged SAP54 (leaf-like flowers) compared to GFP control plants (wild-type flowers) (*t*_(7)_ = 6.45; *p* = 0.008). In these experiments, 10 male and 10 female *M. quadrilineatus* adults were released in a choice cage containing two test plants for the period of 5 d. After removal of adult insects, plants were bagged individually and incubated for 14 d to allow nymph emergence. The graphs in panel A and B represent the percentage of *M. quadrilineatus* nymphs found on each test plant within a single choice cage.

We hypothesized that AY-WB may induce leaf-like flowers as a means of attracting its insect vector, which feeds from the phloem of vegetative tissues. We conducted choice experiments in which *M. quadrilineatus* adults (10 males and 10 females) were released in the middle of a confined space (Figure S7) and were allowed free access to AY-WB–infected *rad23BD* (leaf-like flowers) and *rad23BCD* (non-leaf-like flowers) plants (Figure 5A). Insect preference was then assessed by counting the number of nymphs produced from eggs oviposited on individual plants. Insects produced more progeny on infected *rad23BD* plants versus infected *rad23BCD* plants (*t*_(5)_ = 4.7; *p* = 0.042; Figure 5A), supporting a hypothesis that plants with leaf-like flowers may be more attractive hosts for leafhopper ovipositing. No differences in leafhopper progeny numbers were observed between healthy *rad23BD* and *rad23BCD* mutants (*t*_(5)_ = 0.45; *p* = 0.694; Figure 5A), indicating that insects do not exhibit a preference for either T-DNA mutant line in the absence of AY-WB infection.

We likewise performed leafhopper choice assays with transgenic Arabidopsis lines expressing *35S:GFP* (wild-type flowers) or *35S:GFP–SAP54* (leaf-like flowers) and determined that *M. quadrilineatus* females preferentially oviposit on transgenic lines that express GFP–SAP54 (*t*_(7)_ = 6.45; *p* = 0.008; Figure 5B). As the transgenic lines were not infected with AY-WB, these results clearly indicate that SAP54 has the ability to modulate plant–insect interactions, even in the absence of any additional phytoplasma proteins, thereby promoting AY-WB fitness by enhancing leafhopper vector colonization and oviposition.

Lastly, we wished to assess the contribution of green leaf-like flowers to insect preference in the absence of both AY-WB and SAP54. We thus performed leafhopper choice experiments in which insects were allowed access to Col-0 (wild-type) plants and *ap1* mutants that produce leaf-like flowers with similarity to mild SAP54-expressing transgenic lines (Figure S8). Interestingly, the insects did not exhibit a preference, producing equivalent progeny on Arabidopsis wild-type plants and *ap1* mutants (*t*_(11)_ = 0.22; *p* = 0.835; Figure S9). The developmental defects in *ap1* mutant flowers (which lack petals but are nonetheless fertile flowers that produce seed) may not be severe enough to enhance the attractiveness to the insects. Most likely, SAP54-mediated destabilization of additional MTFs (for example, the SEP paralogues and SOC1) is required for the conversion of all floral whorls into leaf-like organs with increased vegetative biomass, which may be important to modulating plant–insect interactions, or SAP54 may promote insect colonization via a mechanism that extends beyond the growth of leaf-like flowers (for example, modulation of phytohormones). Taken together, our data indicate a role of phytoplasma effector protein SAP54 and Arabidopsis RAD23 in flower development and plant defense to insects.

## Discussion

MTFs comprise a key family of eukaryotic transcription factors that occupy central positions in floral development, dictating both the transition to flowering and the formation of floral organs in all angiosperms [22]. The function of MTFs has been the focus of much study, and these proteins have no known role in plant defence. Similarly, RAD23 proteins are broadly conserved among eukaryotes and play essential roles in mediating the turnover of plant proteins, as evidenced by the lethality of the quadruple *rad23ABCD* Arabidopsis mutant [21]. Microbial effectors functionally characterized so far target specific processes within defence-related pathways, whereas the phytoplasma effector SAP54 has uniquely evolved to link two key pathways involved in plant reproduction and basic protein regulatory processes to alter host development and shape, and thus represents a highly unusual protein. The advantage of targeting conserved host proteins may be particularly relevant for AY-WB, which has a broad plant host range and where the selection of a host is made by the insect vector and not by the phytoplasma. By employing effectors that target conserved (sub)families of plant proteins, AY-WB increases the likelihood that it can modulate developmental processes in the plant species chosen by the insect.

Green leaf-like flowers are a hallmark trait of phytoplasma-infected plants, and we propose that this characteristic trademark is the result of an effector-mediated destabilization of conserved MTFs (i.e., posttranslational regulation via proteolysis). AP1 (with CAL and FUL) and the four SEP proteins play prominent roles in the establishment of a floral meristem and in regulating floral organ identity in the majority of flowering plants [16],[23]. Moreover, SEP3 occupies a central position in mediating the higher order protein interactions necessary to form MTF regulatory complexes [24]. Destabilization of SEP3 (and AP1) by SAP54 is expected to significantly impair the establishment of the MTF protein complexes that are necessary to regulate floral development, especially if accompanied by the destabilization of SEP1, SEP2, and SEP4. Whereas there is much evidence that the expression of various MTFs is mis-regulated in phytoplasma-infected plants [25]–[27], it is unlikely that phytoplasma are directly targeting gene expression. Indeed, the complex auto-regulatory and cross-regulatory network by which MTF gene expression is regulated dictates that the destabilization of several MTFs within this network will result in aberrant gene expression. We have identified several putative SAP54 homologues in other phytoplasma strains (Figure S10), thus indicating that SAP54 may be a member of a larger effector family that modulates floral development.

Animals such as insects also encode MTFs, although a classical Keratin-like domain is absent in these proteins [14]. As an insect-vectored pathogen of plants, AY-WB infects and colonizes both plants and animals. However, the relationship that exists between the phytoplasma and these two hosts is not the same. Phytoplasma are reliant upon their insect vectors for dispersal in the environment, and the association between the phytoplasma and the insects can be beneficial to both organisms [7]. In contrast, phytoplasma are aggressive pathogens of plants, and exposure of a susceptible plant to phytoplasma eventually leads to the death of the plant. Accordingly, SAP54 may have evolved to bind to the plant specific Keratin-like domain of Type II MTFs as a means of selectively targeting plant MTFs and not insect MTFs, which might have a deleterious effect upon AY-WB's vector. Consistent with this hypothesis is the observation that SAP54 expression is up-regulated in plants compared to insects, and many other genes encoding candidate AY-WB effectors show a host-specific expression [9]. Thus, phytoplasmas are likely to produce insect- and plant-specific effectors, in line with their life cycle involving alternate hosts.

SAP54 may escape degradation via the host UPS, as evidenced by the lack of an increase in SAP54 protein levels in epoxomicin-treated tissues (Figure 2C). RAD23 resists proteasomal degradation and is released from the UPS to bind other cargo [28]. It is possible that SAP54 resists degradation by associating with RAD23. SAP54 may simultaneously interact with MTFs and RAD23 upon which the MTFs are delivered to the UPS for degradation, whereas SAP54–RAD23 complexes are released to bind more MTFs (Figure S11A). Although we did not observe ubiquitylation of SAP54 and MTFs in the proteomics analyses, we cannot exclude the possibility that RAD23 interacts with ubiquitin groups linked via lysine (K) residue(s) on SAP54 (Figure S11B). A third possibility is that another pathway is involved in transportation of SAP54–MTF complexes to the host UPS, whereupon SAP54 interacts with RAD23 to evade degradation (Figure S11C). Future research is focused on dissecting these possibilities and the characterization of other plant proteins that are involved in the SAP54-mediated degradation of MTFs in a RAD23-dependent manner. To our knowledge, this is the first report of a microbial effector that recruits RAD23 proteins to enable the inappropriate degradation of a host protein. Nevertheless, exploitation of the UPS by microbial effectors is rapidly emerging as a common theme in plant–pathogen interactions [29]–[31]. Pathogen effectors that suppress, stabilize, or mimic the activity of host ubiquitin ligases have been described [32]–[36], whereas other effectors act as deubiquitylases that catalyze the removal of ubiquitin tags from host proteins [37],[38]. Pathogens also exploit the host UPS to regulate the degradation, localization, and activity of their own effectors via ubiquitination of these proteins, or even by using ubiquitin as a cofactor [39]–[41]. Along this line, RAD23 has been shown to interact with a *Pseudomonas syringae* effector, HopM1 [42], suggesting that RAD23 may have a role in mediating defense-related processes in other host–microbe interactions as well. Taken together, our study extends these earlier observations to reveal that pathogens also exploit host machinery (i.e., shuttle proteins) that is accessory to the UPS.

Insect choice assays involving both AY-WB–infected *rad23* mutants and GFP(-SAP54) lines yield data in support of a hypothesis that leaf-like vegetative flowers are attractive to the phytoplasma leafhopper vector. Our study further demonstrates that SAP54 promotes the colonization of leafhoppers in a RAD23-dependent manner, however it is likely that the RAD23 proteins only play a supporting role in modulating leafhopper behaviour, as leafhoppers do not appear to discriminate between healthy *rad23BD* and *rad23BCD* mutants. On the other hand, experiments performed with Arabidopsis *ap1* mutants suggest that the mere presence of green flowers may not be sufficient to promote leafhopper colonization. Whereas flowers of *ap1* mutants to some extent resemble flowers of AY-WB–infected and SAP54-expressing plants (an observation that is consistent with AP1 destabilization via SAP54), our data strongly indicate that SAP54 targets multiple MTFs for destabilization, including SEP3 and its paralogues. It is thus conceivable that the loss of these MTFs in addition to AP1 is necessary to strongly promote leafhopper colonization. An intriguing (and not mutually exclusive) possibility is that SAP54-mediated promotion of leafhopper colonization extends beyond the recruitment of leaf-like flowers as a means of attracting insects. Whereas leafhoppers may be visually attracted to the bushy, leaf-like appearance exhibited by *35S:GFP–SAP54* plants (Figure S12), we note that AY-WB–infected *rad23BD* mutants do not appear bushier than infected *rad23BCD* mutants, suggesting that “bushiness” is also unlikely to be the sole factor for the observed leafhopper colonization preference. Notwithstanding the above, our study demonstrates convincingly that SAP54 degrades MTFs in a RAD23-dependent manner, resulting in the production of leaf-like flowers and that SAP54 also promotes attractiveness of plants for leafhopper colonization in RAD23-dependent manner. Thus, AY-WB employs at least two protein effectors (SAP54 and SAP11 [8]) that make the plant more suitable for colonization by the insect vector. This is important, because phytoplasmas depend on leafhoppers for transmission and more leafhopper vector progeny will likely result in higher phytoplasma transmission and dispersal rates. Phytoplasma-infected plants are often sterile, as leaf-like flowers do not yield seed, and thus exposed plants become converted into hosts that only serve to help the phytoplasmas reproduce and propagate (zombie plants).

## Materials and Methods

### Yeast Two-Hybrid Analyses

Hybrigenics Services SAS (Paris, France) performed the initial yeast two-hybrid screen, using SAP54 (amino acids 34–124; lacking the signal peptide) cloned into pB27 bait plasmid, as a C-terminal fusion to LexA (N-LexA-SAP54-C). Preliminary testing revealed that SAP54 was not toxic to yeast and did not autoactivate the system. Two screens were performed against a random-primed *Arabidopsis thaliana* seedlings cDNA library constructed into pP6 prey plasmid. A total of 71.7 million clones (7-fold library coverage) were screened following a mating approach with Y187 (matα) and L40 Gal4 (mata) yeast strains as previously described [43]. Of the proteins identified in the Hybrigenics two-hybrid screen and listed in Table S1, we independently confirmed the interaction of each protein with SAP54 using yeast strain MaV203 (Invitrogen) with plasmids pDEST32 (GAL4–DNA-binding domain) and pDEST22 (GAL4-activation domain) as follows. MaV203 was transformed according to [44], and transformants were selected and maintained by growth on minimal medium lacking leucine (to select for pDEST32) and tryptophan (to select for pDEST22). To examine protein–protein interactions, freshly transformed yeast colonies were resuspended in 1 mL sterile deionized water, and 10 µL aliquots were spotted upon medium lacking leucine and tryptophan (−L/−W) and medium lacking leucine, tryptophan, histidine, supplemented with 60 mM 3-Amino-1,2,4-triazole (3-AT; Sigma Aldrich) (−L/−W/−H). Growth was scored after 5 to 7 d of incubation at 28°C.

For the comprehensive MTF yeast two-hybrid assay, a matrix-based approach was followed as described previously [45]. The originally described GAL4-AD and GAL4-BD MTF collection [11] was extended with a number of known MTF splicing variants [46],[47], making a total of 106 MTF proteins expressed from the pDEST22 and pDEST32 vector. The above-described pDEST32–SAP54 and a pDEST22–SAP54 construct were used as bait, respectively, in the pair-wise screening. Growth of yeast, and hence interaction events, was scored after 5 d of incubation at 20°C on synthetic dropout (SD) medium lacking leucine, tryptophan, histidine, supplemented with 1 mM 3-AT (−L/−W/−H). All identified positives were rescreened in a second experiment, in which the yeast was spotted onto selective medium lacking leucine, tryptophan, and adenine (−L/−W/−A).

For Western blot analysis, we followed a protocol established by Kushnirov [48]. Yeast strains were grown in liquid growth medium lacking leucine and tryptophan at 28°C overnight, and 2.5 OD_600_ of yeast cells were harvested for each experiment.

### Co-Expression and Co-Immunoprecipitation Assays

*SAP54* was amplified using a forward primer that encodes a Flag-tag (Table S10), thus enabling the expression of SAP54 (amino acids 34–122) with an N-terminal Flag-tag in place of its signal peptide. The PCR product was cloned into pTRBO [18] via restriction enzyme sites AvrII and NotI using standard molecular techniques. The construction of Flag-tagged RFP has already been described [8]. Type I and Type II MTFs were initially cloned into pDONR207 using BP Clonase (Invitrogen), and the genes were then transferred into pGWB21 [49] via LR Clonase (Invitrogen) to generate 35S:10xmyc–MTF. Primers used to amplify these genes are included in Table S10. All genes cloned into pDONR207 as a result of this study were sequenced prior to use to ensure the absence of mutations within the gene. Furthermore, we also sequenced the gene insert within the 10xmyc-tagged MTF plasmids following completion of our co-expression assays to ensure the validity of our plasmids.

Co-expression assays were performed using constructs transiently expressed in *Nicotiana benthamiana* leaves of 3- to 4-wk-old plants that were grown in a controlled growth room in 16/8-h light/dark at 22°C with 55% humidity. 35S:10xmyc-tagged MTFs and 35S:Flag-tagged RFP or SAP54 were expressed in parallel following agroinfiltration using a needless syringe, using a 1∶1 mixture of *Agrobacterium tumefaciens* (strain GV3101) cultures adjusted to a final OD_600_ of 0.1–0.2 (RFP and SAP54) and OD_600_ of 0.5 (MTFs). For each co-expression assay, one leaf per plant (two plants per assay) was agroinfiltrated, with the Flag-RFP + Myc-MTF mixture infiltrated on the left side and Flag-SAP54 + Myc-MTF mixture infiltrated on the right side of the same leaf. After 3 d, one leaf disk (11 mm diameter) per infiltrated area was harvested and these were frozen immediately in liquid nitrogen. For Western blot analysis, two frozen leaf disks per sample were ground using a mortar and pestle in liquid nitrogen. We added 30 µL of 4× NuPage LDS sample buffer (Invitrogen) to the powdered tissue, and samples were boiled for 10 min. In a typical co-expression assay, 3 µL (anti-Flag blot), 5 µL (anti-SAP54 blot), and 15 µL (anti-Myc blot) aliquots were loaded into the SDS-PAGE gel.

Epoxomicin-treated samples were agroinfiltrated as described above, however 50 µM epoxomicin (Merck Chemicals LTD) was infiltrated using a needless syringe into the relevant area 3 d after agroinfiltration. The epoxomicin was prepared immediately prior to use by adding 2.78 µL of a 18 mM stock (dissolved in 100% DMSO) into 1 mL of sterile water. An equal volume of 100% DMSO (Sigma Aldrich) was added to sterile water to comprise the DMSO-only control, which was infiltrated alongside the epoxomicin treatment. Leaf disks from epoxomicin-treated (and DMSO-treated) samples were harvested after 8 h, and leaf disks were frozen in liquid nitrogen.

For co-immunoprecipitation assays performed using protein transiently expressed in *N. benthamiana* leaves, agroinfiltration was performed as describe above, however two entire leaves were infiltrated (per construct) to provide sufficient material (typically yielding about 1–2 grams of tissue). Leaf disks were removed from each leaf prior to freezing in liquid nitrogen, and Western blots were performed using these disks to confirm adequate protein expression levels prior to co-immunoprecipitation experiments. Following this verification, the remaining sample was ground using a mortar and pestle in liquid nitrogen, and added to cold extraction buffer (150 mM Tris-HCl, pH 7.5, 150 mM NaCl, 10% glycerol, 10 mM EDTA, 20 mM sodium fluoride, 10 mM DTT, 0.5% (wt/v) polyvinylpolypyrrolidone, 0.1% Triton-X, protease cocktail inhibitor (Sigma Chemical)) on ice. Samples were centrifuged at 3,200× *g* at 4°C for 15 min, and the supernatant was filtered through a 0.45 µm filter (Sartorius Stedim UK Limited) using a needleless syringe. We added 2 mL filtered extract to 20 µL equilibrated GFP-binding affinity resin (GFP-Trap_M; Chromotek GMBH), and samples were incubated at 4°C overnight upon a rotating wheel. Samples were initially pelleted by centrifugation at 2,700× *g* for 2 minutes and pellets were washed with 1 mL TBS buffer (10 mM Tris-HCl, pH 7.5, 150 mM NaCl, 0.5 mM EDTA, 0.1% Tween-20). Subsequent wash steps were performed using a magnetic stand to pellet GFP-binding resin. Samples were washed a minimum of three times, and all steps were performed at 4°C using ice-cold buffer. Following the final wash, all buffer was carefully removed using a syringe fitted with a 27G needle, and the resin was resuspended in 20 to 30 µL 4× NuPage LDS sample buffer (Invitrogen) and boiled for 10 min prior to loading on SDS/PAGE gels. In a typical co-immunoprecipitation experiment, 1 to 3 µL (anti-GFP) and 10 to 15 µL (anti-myc) aliquots were loaded onto an SDS-PAGE gel.

Co-immunoprecipitation assays performed using Arabidopsis transgenic lines were performed as described above with a few modifications. Transgenic lines expressing GFP-tagged AG, FUL, and SEP3 under control of their native promoters are described in [15],[50]. To minimize loss of MTFs due to SAP54-mediated destabilization, flowers were collected at an early stage of infection approximately 2–3 wk following exposure to noncarrier (for healthy flowers) and AY-WB–carrier (for infected flowers) *Macrosteles quadrilineatus*. At this point, plants are only beginning to produce flowers and early flowers appear normal or exhibit a mild degree of phyllody. Flowers produced at a later stage of infection (4 wk following inoculation) exhibit a strong degree of phyllody and loss of determinacy (samples of these flowers were harvested for Western blot analysis in Figure 2A). For mass spectrometry, samples were collected from stably (T1) transformed Arabidopsis lines expressing either *35S:GFP–SAP54* or *35S:GFP* as a control. Primary and secondary inflorescences were harvested comprising all stages of developing floral buds from plants grown in a long day photoperiod (16/8-h light/dark). We used 0.85 to 0.90 g of plant tissues per pull-down experiment, and immunoprecipitation was performed using 50 µL equilibrated GFP-binding affinity resin. The GFP-binding resin was resuspended in 45 µL 4× NuPage LDS sample buffer prior to boiling. Western blots (anti-GFP) were performed to confirm the successful immunoprecipitation of GFP–SAP54 (or GFP) using 2 µL aliquots of each sample prior to further analysis via mass spectrometry. The remaining sample (approximately 40 µL) was resolved upon a 1.5 mm NuPAGE 4–12% Bis-Tris gel (Invitrogen) using a MOPS SDS running buffer. Proteins were visualized using SimplyBlue SafeStain (Invitrogen) and protein bands were cut out and collected using a new razor blade. In areas of the lane with no visible protein, 10 mm×10 mm gel slices were collected. All gel slices were destained in 30% ethanol (3×30 min washes at 65°C) prior to mass spectrometry analysis.

### Western Blots

Proteins were separated on 12.5% (wt/v) SDS-PAGE gels and transferred to 0.45 µm Protran BA85 nitrocellulose membranes (Whatman) using the BioRad minigel and blotting systems following standard protocols. Blotted membranes were incubated in blocking buffer (5% (wt/v) milk powder in 1× phosphate buffered saline and 0.1% (v/v) Tween-20) with primary antibody at 4°C overnight. Peroxidase-conjugated anti-rabbit or anti-mouse secondary antibody (Sigma Aldrich) was added to washed blots and incubated at room temperature for 4 h. Bound antibodies were detected using Immobilon Western Chemiluminescent HRP Substrate (Millipore). Protein loading was visualized using Ponceau S solution (0.1% (wt/v) in 5% acetic acid; Sigma Aldrich). Signal intensity was quantified by generating density histograms for each protein band and then determining the area within the corresponding peak using ImageJ.

Anti-SAP54 antibodies were raised in rabbits (Genscript) injected with purified 6×His-tagged SAP54. A 1∶2,000 dilution of anti-SAP54 was used in Western blots, and is sufficient to detect <10 ng purified SAP54. Monoclonal anti-Myc, anti-GFP, anti-Flag, and anti-GAL4AD antibodies (all from Sigma Aldrich) were used at a 1∶10,000 dilution. Anti-RAD23 antibodies (used at a 1∶10,000 dilution) were provided by R. D. Vierstra and were raised in rabbit [21]. Anti-SEP3 antibodies (used at a 1∶1,000 dilution) were provided by Cezary Smaczniak and were raised in rabbit.

### Generation of AY-WB–Infected Plants

Healthy and AY-WB–carrier *Macrosteles quadrilineatus* Forbes (Hemiptera: Cicadellidae) stocks were maintained as previously described [8]. To generate infected Arabidopsis, three male AY-WB–carrier leafhoppers were added per plant to 4-wk-old plants that were previously grown in a short day photoperiod (10/14-h light/dark). Insects and plants were left for 4 to 5 d to allow inoculation of the phytoplasma in a controlled growth chamber set to a long day photoperiod to stimulate flowering (16/8-h light/dark). Insects were then removed and plants were returned to the growth chamber. Leaf-like flowers are typically produced 3 wk following initial exposure to the infected leafhoppers. Healthy controls were included in each experiment and comprised plants that were exposed to an equivalent number of noncarrier *Macrosteles quadrilineatus* leafhoppers as infected plants, and insects were removed on the same day as infected plants. The infection status of plants was determined via PCR analysis of DNA extracted from rosette leaves using AY-WB–specific primers BR and BF (Table S10) [51].

### Insect Choice Assays

*rad23BD* and *rad23BCD* lines are described in [21], whereas seed for the *ap1* mutant was obtained from NASC (ID: N6232, allele *ap1-12*). Plants were sown on insecticide-free F2 compost soil (Levington) and grown at 22°C in a growth chamber adjusted to a short day photoperiod (8/16-h light/dark). Five-week-old plants were infected with AY-WB by adding five male AY-WB–carrier *Macrosteles quadrilineatus* leafhoppers to each plant in a transparent perspex tube (10 cm high, diameter 4 cm) for 5 d. Test plants were transplanted in 10 cm×10 cm square pots (F2 soil) and grown for an additional 2 wk at 22°C in a growth chamber adjusted to a long day photoperiod (16/8-h light/dark) to stimulate flowering. Prior to choice experiments, three rosette leaves were collected for extraction of genomic DNA to confirm the genotype of all plants (using primers as previously described [21]) and AY-WB infection status (using AY-WB–specific primers BF and BR in Table S10).

All insect choice experiments were performed in transparent polycarbonate cages 620 mm×300 mm×410 mm (height×width×length). Two opposite sides of the cage were fitted with white nylon mesh held in place by magnetic strips to enclose the cage. Two test plants (21 dai with AY-WB) were randomly placed diagonally opposite each other in the corners of a cage (Figure S7). Ten male and 10 female healthy adult *M. quadrilineatus* leafhoppers were released from a transparent perspex tube (9 cm high, diameter 3 cm) in the centre of the cage equidistant from each test plant. Adult insects were removed 5 d after addition to the cage. At that time, plants were removed from the choice cage and enclosed individually in transparent perforated plastic bags, and returned to the growth chamber. Nymphs were counted on each test plant 14 d after removal of adult insects from the cages. Data were expressed as proportion of total number of nymphs found on the test plants within each choice cage.

### Statistical Analysis

Statistical analysis was performed in Minitab16. Insect oviposition choice data were analysed using paired *t* test. Assumptions of the statistical tests (normal distribution and equal variance) were checked with the Anderson-Darling and the Levene's tests, respectively.

### Generation of *35S:GFP–SAP54* and *35S:GFP* Transgenic Arabidopsis Lines

The gene encoding SAP54 (lacking the signal peptide; amino acids 34 to 124) was PCR-amplified using primers attB1SAP54 and attB2SAP54 (Table S10) and cloned in pDONR207 (Invitrogen) using Clonase BP according to the manufacturer's instructions. For expression in Arabidopsis, *SAP54* was transferred into Gateway vector pB7WGF2 using Clonase LR as per the manufacturer's instructions. pB7WGF2 encodes an N-terminal GFP fragment under control of the CaMV 35S promoter [52], thus generating *35S:GFP–SAP54*. For the construction of a *35S:GFP* transgenic Arabidopsis line, the gene encoding eGFP was amplified using pB7WGF2 as a template and primers attB1foreGFP and attB2reveGFP (Table S10). *eGFP* was then cloned into pDONR207 using Clonase BP, and the gene was transferred into pB7WG2 [52] to create *35S:GFP*. Arabidopsis plants were transformed via floral dip [53] with *Agrobacterium tumefaciens* strain GV3101. Seedlings of transformed plants were selected by the herbicide glufosinate (BASTA). Transgenic plants expressing GFP–SAP54 are sterile (with the exception of transformants obtained in *rad23CD* and *rad23BCD* backgrounds); thus, experiments and phenotypic analyses were performed upon T1 lines. Prior to the assessment of GFP–SAP54–induced phenotypes in *rad23* T-DNA mutant lines, a minimum of 10 randomly selected plants from each transformation group were examined via PCR analysis, using primers specific to each of the four *RAD23* genes as described in [21] to confirm the genotype of the plants. *rad23* single mutants are the result of T-DNA insertions (SALK lines 066603, 075940, 068091, and 014137) and higher order mutants were kindly provided by Richard Vierstra and are described in [21].

### Mass Spectrometry Analysis

Gel slices cut from the SDS-PAGE gel were washed, reduced and alkylated, and treated with trypsin according to standard procedures [54]. Peptides were extracted with 5% formic acid/50% acetonitrile, dried down, and re-dissolved in 0.1% TFA. For LC-MS/MS analysis, a sample aliquot was applied via a nanoAcquityTM (Waters, Manchester, UK) UPLCTM-system running at a flow rate of 250 nL min-1 to an LTQ-Orbitrap mass spectrometer (Thermo Fisher, Waltham, MA). Peptides were trapped using a pre-column (Symmetry C18, 5 µm, 180 µm×20 mm, Waters) that was then switched in-line to an analytical column (BEH C18, 1.7 µm, 75 µm×250 mm, Waters) for separation. Peptides were eluted with a gradient of 3–38% acetonitrile in water/0.1% formic acid at a rate of 0.67% min-1. The column was connected to a 10 µm SilicaTip nanospray emitter (New Objective, Woburn, MA, USA) attached to a nanospray interface (Proxeon, Odense, Denmark) for infusion into the mass spectrometer. The mass spectrometer was operated in positive ion mode at a capillary temperature of 200°C. The source voltage and focusing voltages were tuned for the transmission of MRFA peptide (m/z 524) (Sigma Aldrich, St. Louis, MO). Data-dependent analysis was carried out in oribtrap-IT parallel mode using CID fragmentation on the five most abundant ions in each cycle. The orbitrap was run with a resolution of 30,000 over the MS range from m/z 350 to m/z 1800 and an MS target of 106 and 1 s maximum scan time. Collision energy was 35, and an isolation width of 2 was used. Only mono-isotopic 2+ and 3+ charged precursors were selected for MS2. The MS2 was triggered by a minimal signal of 1,000 with an AGC target of 3×104 ions and 150 ms scan time using the chromatography function for peak apex detection. Dynamic exclusion was set to 1 count and 30 s exclusion with an exclusion mass window of ±20 ppm. MS scans were saved in profile mode, whereas MSMS scans were saved in centroid mode.

Raw files were processed with MaxQuant version 1.3.0.5 ([55]; http://maxquant.org) to generate re-calibrated peaklist-files which were used for a database search using an in-house Mascot 2.4 Server (Matrix Science Limited, London, UK). Mascot-mgf files were generated from MaxQuant apl-files using a suitable perl script. Mascot searches were performed on the TAIR_10_pep_20101214.fasta database (http://www.arabidopsis.org/) using trypsin/P with 2 missed cleavages, 6 ppm precursor tolerance, 0.6 Da fragment tolerance, carbamidomethylation (C) as fixed, and oxidation (M) and acetylation (protein N-terminus) as variable modifications. Mascot search results were imported and evaluated in Scaffold 4.0.4 (proteomsoftware.com, Portland, OR, USA) resulting in a protein false discovery rate of 0.9%.

## Supporting Information

Figure S1 **Analysis of SAP54 interactions with MADS-domain proteins.** (A) Type I MTFs AGL50, AGL62, and AGL80 are partially destabilized when transiently co-expressed in the presence of the phytoplasma effector SAP54. (B) Noncanonical Type II MIKC* protein AGL66 is stable in the presence of SAP54. (C) SAP54-mediated destabilization of Type II MIKC^C^ protein AP1 is inhibited following treatment with 50 µM epoxomicin, whereas AP1 is destabilized in samples treated with a protease inhibitor cocktail. (D) AP1, SEP3, and SOC1 are not destabilized by SAP54 in yeast.
(TIFF)

Figure S2 **Phenotype of various** ***rad23*** **mutants.** (A) Four-week-old Arabidopsis wild-type (Col-0) and *rad23* mutant lines. Note the reduced stature of *rad23BC*, *rad23BD*, *rad23ABD*, and *rad23BCD* plants. Scale bar, 5 cm. (B) *rad23* mutants produce wild-type flowers, with the exception of the *rad23BCD* triple mutant that frequently produces flowers with five or six petals (lateral and frontal view as shown).
(TIFF)

Figure S3 **Scoring of phenotypes exhibited by** ***35S:GFP–SAP54*** **transgenic lines.** Plants were scored as follows, with representative flowers depicted. Absent, flowers are indistinguishable from wild-type based upon visual examination. Mild, enlarged sepals, mild to moderate virescence of petals, stamens produce pollen, and occasional loss of determinacy observed in early arising flowers. Strong, leaf-like sepals, strong virescence of petals, stamens are virescent and do not produce pollen, frequent loss of determinacy throughout the plant.
(TIF)

Figure S4 **Characterization of transgenic Arabidopsis lines expressing** ***35S:GFP–SAP54*** **.** (A) Expression of *GFP–SAP54* induces phyllody and loss of determinacy in wild-type Arabidopsis Col-0, but the majority of transformants obtained in *rad23CD* double mutants produce normal flowers. *35S:GFP–SAP54 rad23CD* line 8 represents a minority of transgenic lines exhibiting a mild degree of virescence and loss of determinacy. (B) Western blot analysis reveals protein levels of GFP–SAP54 (indicated by an arrow) in transgenic plants picture in panel A.
(TIFF)

Figure S5 **AY-WB phytoplasma induces witches' broom but not phyllody in infected** ***rad23BCD*** **triple mutants.** (A) An image of a healthy (wild-type) Arabidopsis plant. (B) Wild-type (Col-0) and *rad23* triple mutants following infection with AY-WB phytoplasma. Note the occurrence of witches' broom (increased proliferation of stems) in all plants. (C) Wild-type (Col-0) and *rad23ABD* produce leaf-like flowers when infected with AY-WB, whereas the *rad23BCD* mutant produces normal flowers.
(TIFF)

Figure S6 **Arabidopsis** ***rad23*** **mutants produce leaf-like flowers following infection with phytoplasma AY-WB.**
(TIF)

Figure S7 **Experimental set-up for insect oviposition choice experiments.** Photograph (top) illustrates the actual arrangement of the test plants (A, D) in a choice cage. Several other choice cages are visible in the background with alternative positioning of the test plants. Diagramme (bottom) depicts all available positions for the test plants in the cage (A, B, C, D). Only two positions are occupied in any given cage, resulting from randomly placing the test plants in two out of the four available corners. Insects are introduced in the center of the cage (equidistant from both plants) and released from a transparent plastic tube (E). Arrows indicate the physical dimensions of the cage.
(TIFF)

Figure S8 **Arabidopsis** ***ap1-12*** **mutants produce green leaf-like flowers that lack petals.** (A) Images of flowers from healthy and AY-WB–infected Arabidopsis wild-type (Col-0) are compared to a GFP–SAP54–expressing transgenic line and *ap1-12* mutant. (B) Images of plants representative of healthy and AY-WB–infected Arabidopsis, GFP–SAP54–expressing transgenic lines, and *ap1-12* mutants. Scale bars, 5 cm. Se, sepal; Pe, petal; St, stamen; Ca, carpel.
(TIFF)

Figure S9 **Aster leafhopper** ***Macrosteles quadrilineatus*** **produces a similar number of nymphs on wild-type (wt) and** ***ap1*** **mutant Arabidopsis plants (** ***t*** **_(11)_ = 0.22;** ***p*** **= 0.835).**
(TIFF)

Figures S10 **Alignment of amino acid sequences of SAP54 homologues identified in other phytoplasma strains.** M-AY, Maryland aster yellows phytoplasma (ABH11652); Spiraea, Spiraea stunt phytoplasma (ABU55747); OY-M, Onion yellows phytoplasma strain OY-M (PAM_049); PnWB, Peanut Witches' Broom phytoplasma (ZP_23918844).
(TIFF)

Figure S11 **Models of SAP54-mediated degradation of MTFs.** (A) SAP54 binds directly to both MTFs and RAD23. The latter takes the SAP54–MTF complex to the plant UPS where the MTFs are degraded. SAP54 may remain associated with RAD23 to prevent being degraded. (B) RAD23 and SAP54 do not interact directly, but via one or more ubiquitin moieties linked via lysine (K) residue(s) on SAP54. RAD23 takes the SAP54–MTF complex to the plant UPS (as in A). (C) An unknown pathway is involved in transportation of SAP54–MTF complexes to the host UPS, whereupon SAP54 interacts with RAD23 to evade degradation. RAD23 and SAP54 may interact directly (as in A) or via ubiquitin (as in B).
(TIFF)

Figure S12 **Phytoplasma effector SAP54 alters host development to promote vegetative growth.** Shown are 7-wk-old (A) and 10-wk-old (B) transgenic Arabidopsis lines expressing *35S:GFP* (control) and *35S:GFP–SAP54*. Scale bars, 5 cm.
(TIFF)

Table S1 **Clones identified as SAP54 interactors in Hybrigenics screen.**
(DOC)

Table S2 **Yeast two-hybrid analysis of SAP54 interactions with MTFs.**
(DOC)

Table S3 **Quantification of signal intensity levels (ImageJ) of bands in** **Figure 1D** **.**
(DOC)

Table S4 **Mass spectrometry analysis of MTFs that interact with GFP–SAP54.**
(DOC)

Table S5 **Signal intensity levels (ImageJ) of bands in** **Figure 2A** **.**
(DOC)

Table S6 **Signal intensity levels (ImageJ) of bands in** **Figure 2B** **.**
(DOC)

Table S7 **Signal intensity levels (ImageJ) of bands in** **Figure 2C** **.**
(DOC)

Table S8 **Signal intensity levels (ImageJ) of IP bands in** **Figure 2D** **.**
(DOC)

Table S9 **Signal intensity levels (ImageJ) of input bands in** **Figure 2D** **.**
(DOC)

Table S10 **List of primers used in study.**
(DOC)

## Abbreviations

AG: AGAMOUS
AGL: AGAMOUS-LIKE
AP1: APETALA1
AY-WB: Aster Yellows phytoplasma strain Witches' Broom
Col-0: Columbia
FUL: FRUITFULL
GFP: Green fluorescent protein
K-domain: Keratin-like domain
MAF1: MADS AFFECTING FLOWERING1
MTFs: MADS-domain transcription factors
MIKC^C^: canonical MADS-domain proteins
MIKC*: noncanonical MADS-domain proteins
RAD23: RADIATION SENSITIVE23
RFP: red fluorescent protein
SEP: SEPALLATA
SOC1: SUPPRESSOR OF OVEREXPRESSION OF CONSTANS1
UPS: ubiquitin/26S proteasome system
