# Supplementary figures and images for: Phytoplasma Effector SAP54 Hijacks Plant Reproduction by Degrading MADS-box Proteins and Promotes Insect Colonization in a RAD23-Dependent Manner
Source: PLoS Biol. 2014 Apr 8;12(4):e1001835. doi: 10.1371/journal.pbio.1001835 (PMC3979655; doi:10.1371/journal.pbio.1001835)

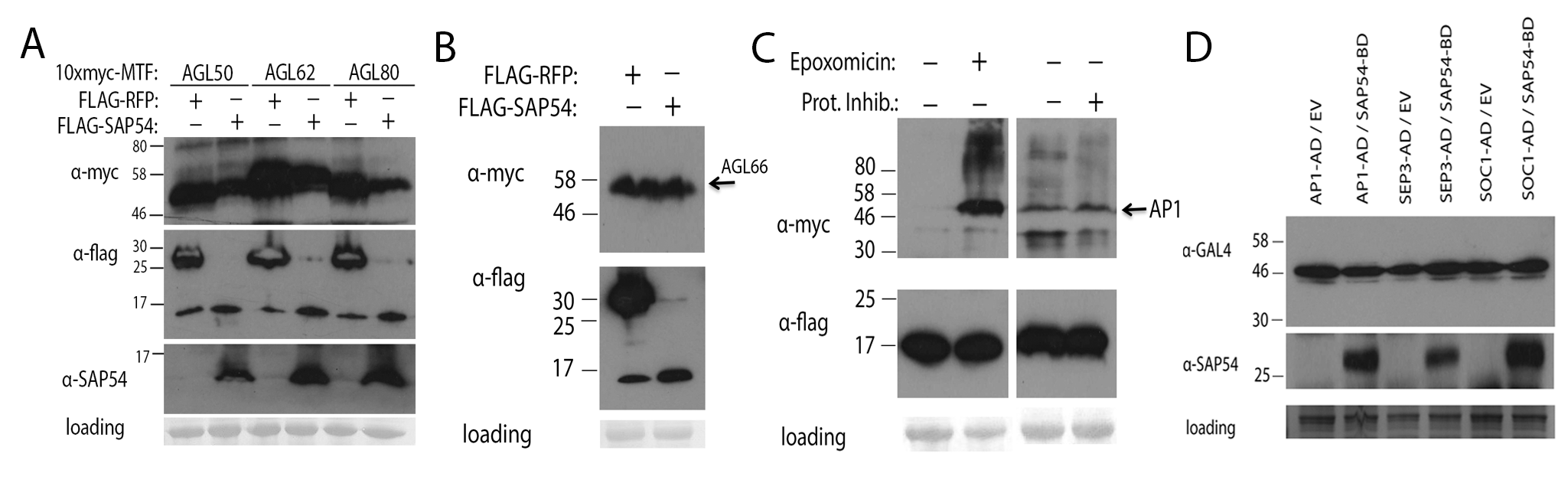

Supplement: Figure S1 — Analysis of SAP54 interactions with MADS-domain proteins. (A) Type I MTFs AGL50, AGL62, and AGL80 are partially destabilized when transiently co-expressed in the presence of the phytoplasma effector SAP54. (B) Noncanonical Type II MIKC* protein AGL66 is stable in the presence of SAP54. (C) SAP54-mediated destabilization of Type II MIKCC protein AP1 is inhibited following treatment with 50 µM epoxomicin, whereas AP1 is destabilized in samples treated with a protease inhibitor cocktail. (D) AP1, SEP3, and SOC1 are not destabilized by SAP54 in yeast. (TIFF) [file pbio.1001835.s001.tiff]

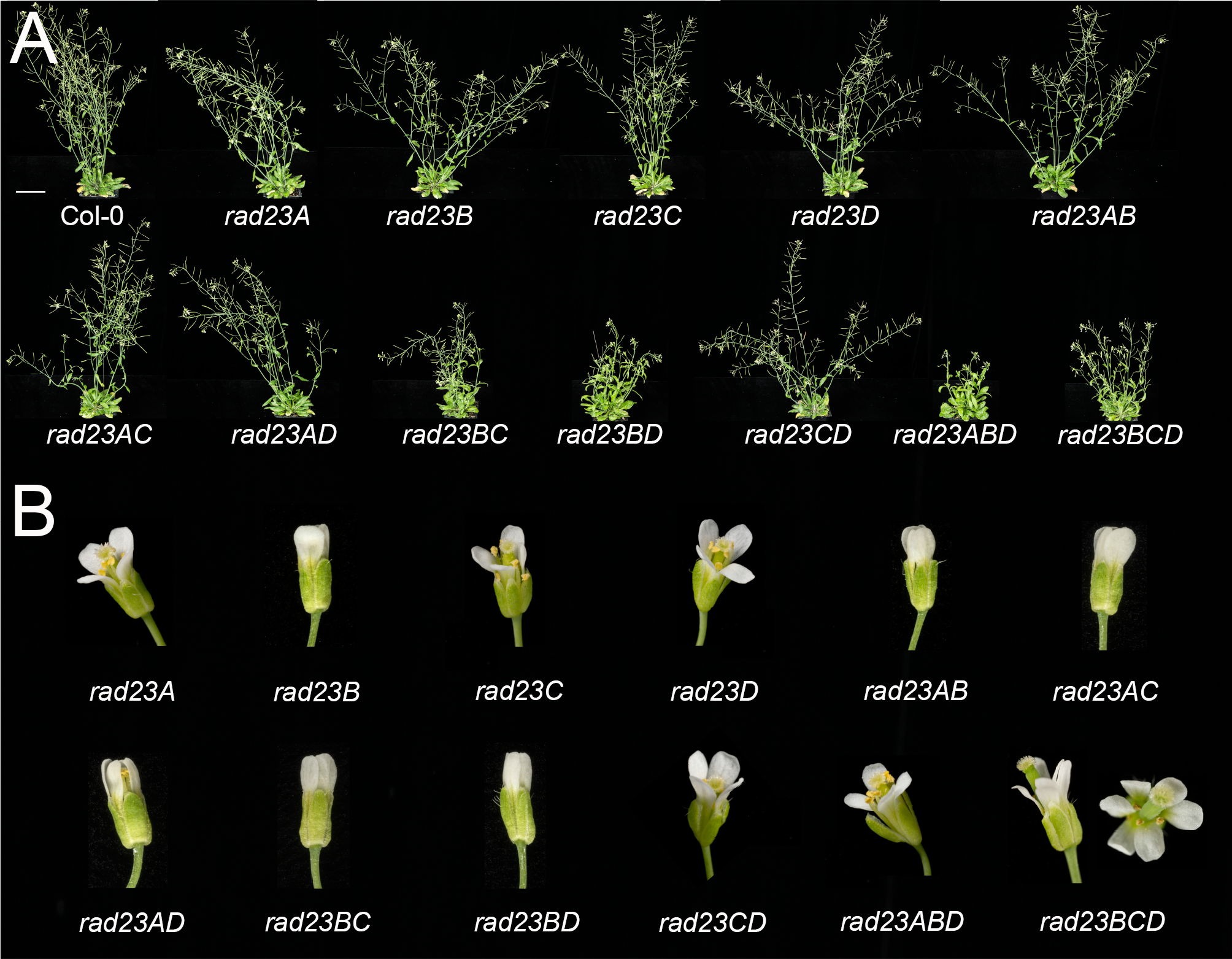

Supplement: Figure S2 — Phenotype of various rad23 mutants. (A) Four-week-old Arabidopsis wild-type (Col-0) and rad23 mutant lines. Note the reduced stature of rad23BC, rad23BD, rad23ABD, and rad23BCD plants. Scale bar, 5 cm. (B) rad23 mutants produce wild-type flowers, with the exception of the rad23BCD triple mutant that frequently produces flowers with five or six petals (lateral and frontal view as shown). (TIFF) [file pbio.1001835.s002.tiff]

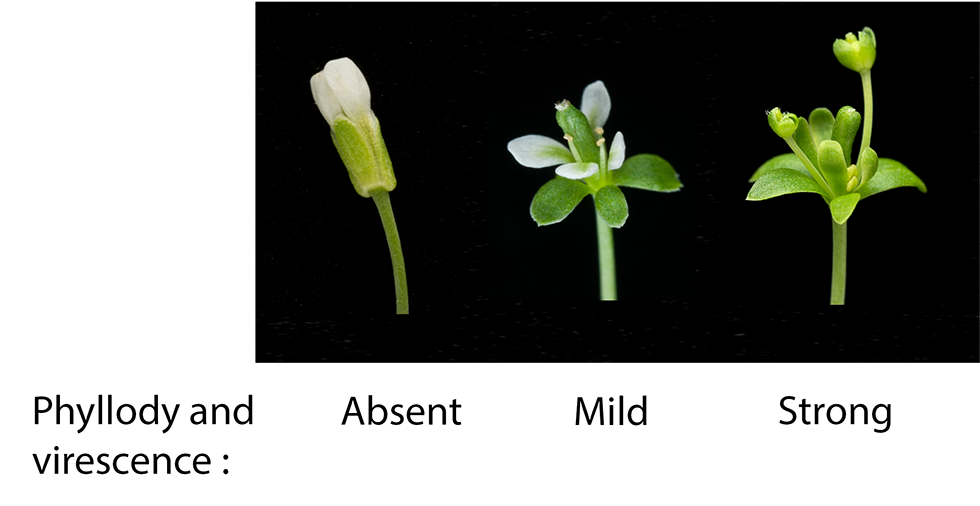

Supplement: Figure S3 — Scoring of phenotypes exhibited by 35S:GFP–SAP54 transgenic lines. Plants were scored as follows, with representative flowers depicted. Absent, flowers are indistinguishable from wild-type based upon visual examination. Mild, enlarged sepals, mild to moderate virescence of petals, stamens produce pollen, and occasional loss of determinacy observed in early arising flowers. Strong, leaf-like sepals, strong virescence of petals, stamens are virescent and do not produce pollen, frequent loss of determinacy throughout the plant. (TIF) [file pbio.1001835.s003.tif]

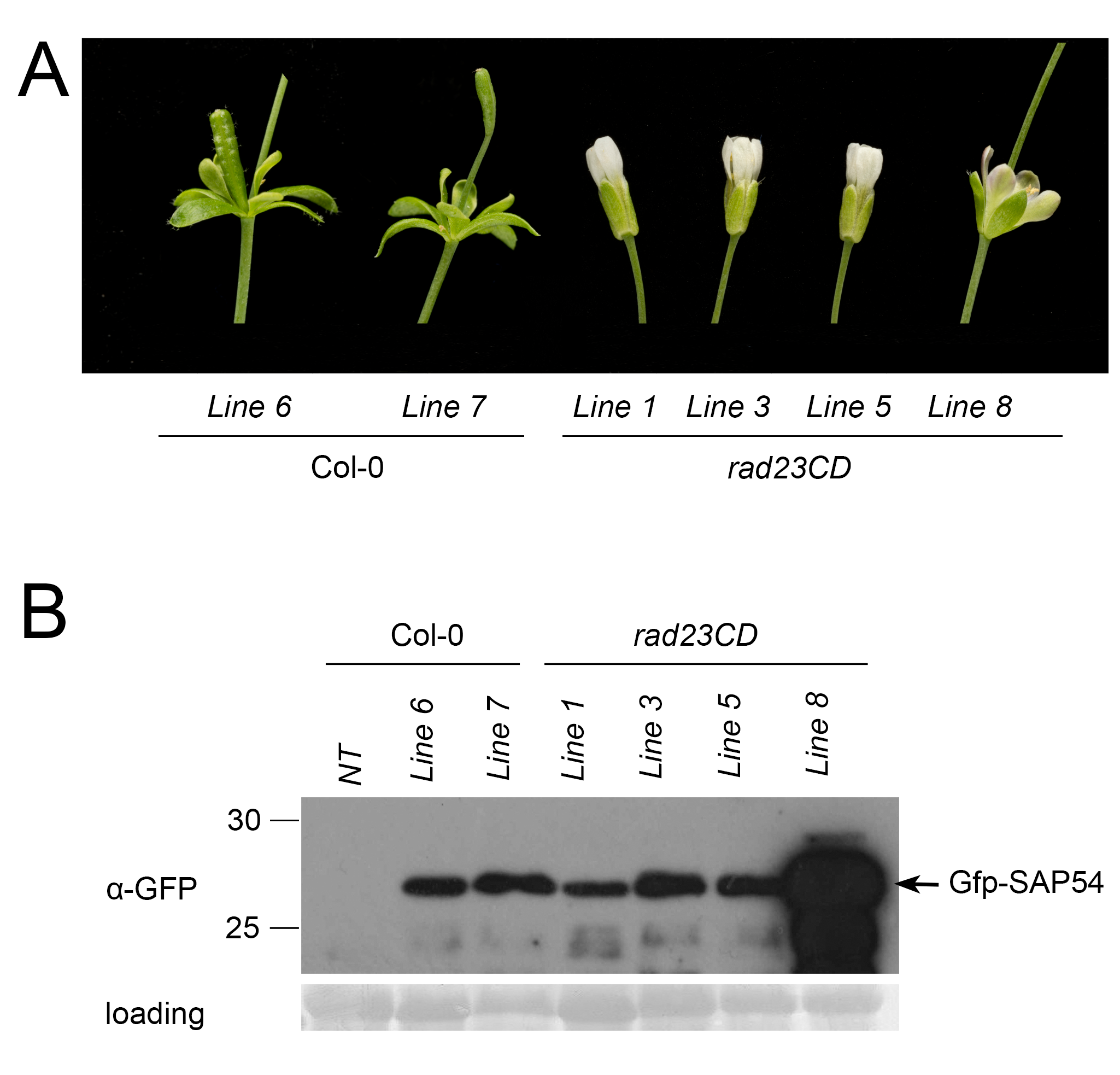

Supplement: Figure S4 — Characterization of transgenic Arabidopsis lines expressing 35S:GFP–SAP54 . (A) Expression of GFP–SAP54 induces phyllody and loss of determinacy in wild-type Arabidopsis Col-0, but the majority of transformants obtained in rad23CD double mutants produce normal flowers. 35S:GFP–SAP54 rad23CD line 8 represents a minority of transgenic lines exhibiting a mild degree of virescence and loss of determinacy. (B) Western blot analysis reveals protein levels of GFP–SAP54 (indicated by an arrow) in transgenic plants picture in panel A. (TIFF) [file pbio.1001835.s004.tiff]

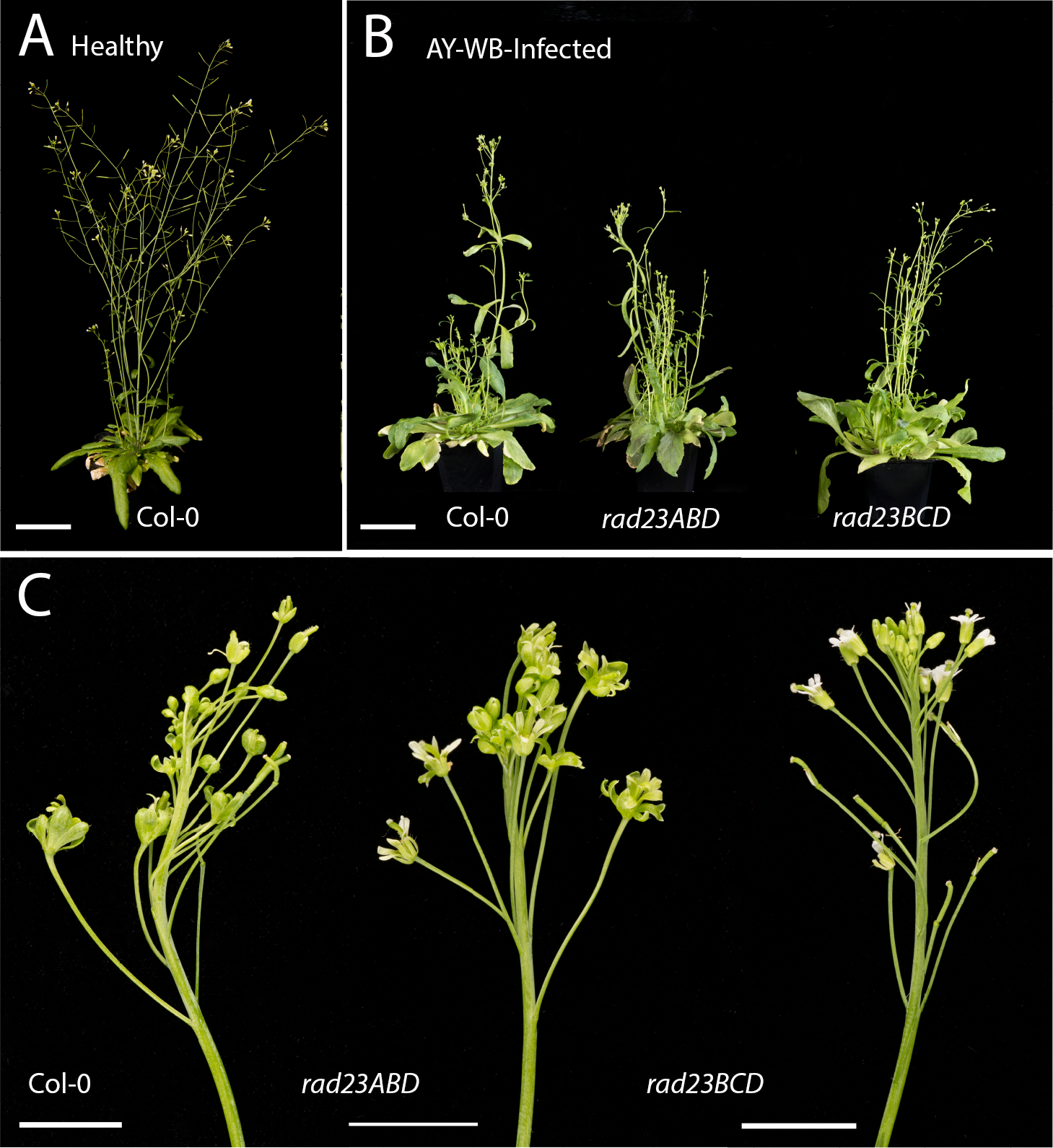

Supplement: Figure S5 — AY-WB phytoplasma induces witches' broom but not phyllody in infected rad23BCD triple mutants. (A) An image of a healthy (wild-type) Arabidopsis plant. (B) Wild-type (Col-0) and rad23 triple mutants following infection with AY-WB phytoplasma. Note the occurrence of witches' broom (increased proliferation of stems) in all plants. (C) Wild-type (Col-0) and rad23ABD produce leaf-like flowers when infected with AY-WB, whereas the rad23BCD mutant produces normal flowers. (TIFF) [file pbio.1001835.s005.tiff]

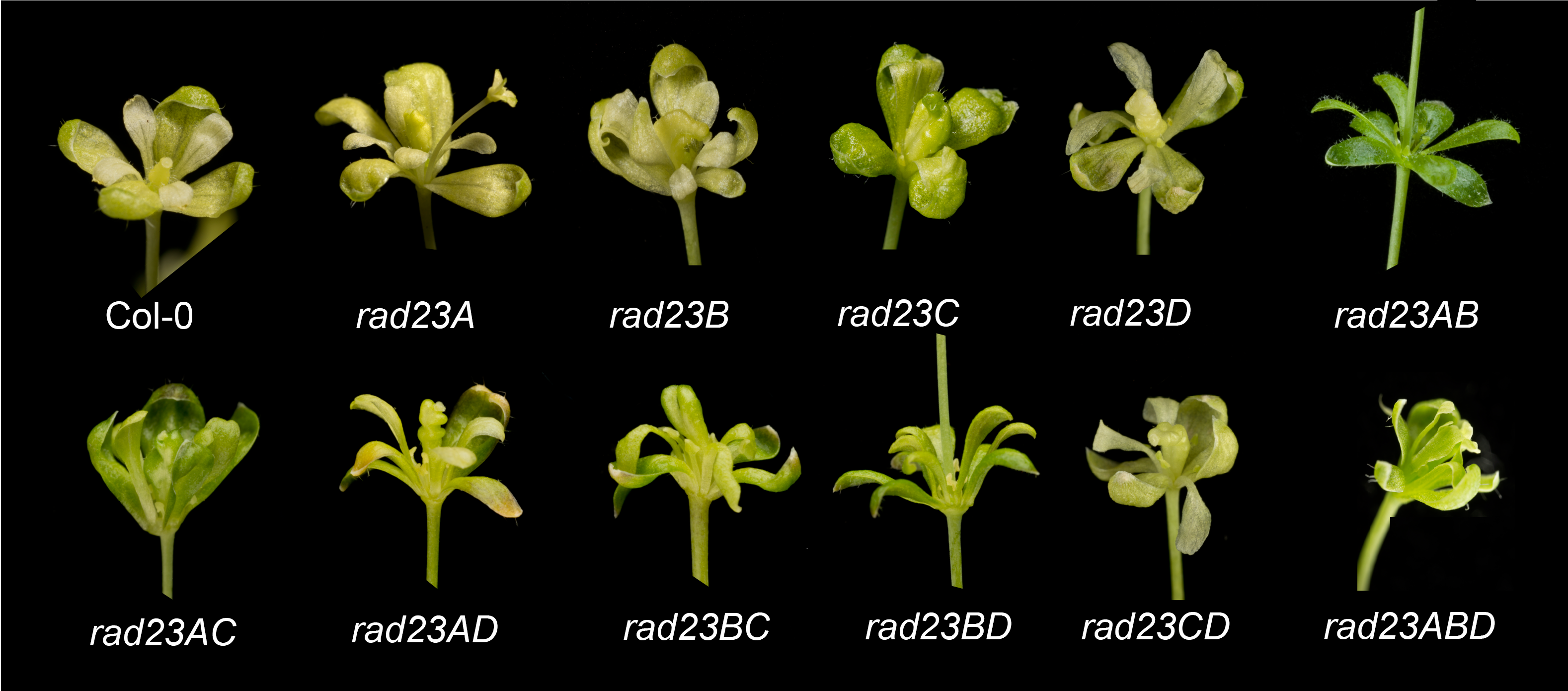

Supplement: Figure S6 — Arabidopsis rad23 mutants produce leaf-like flowers following infection with phytoplasma AY-WB. (TIF) [file pbio.1001835.s006.tif]

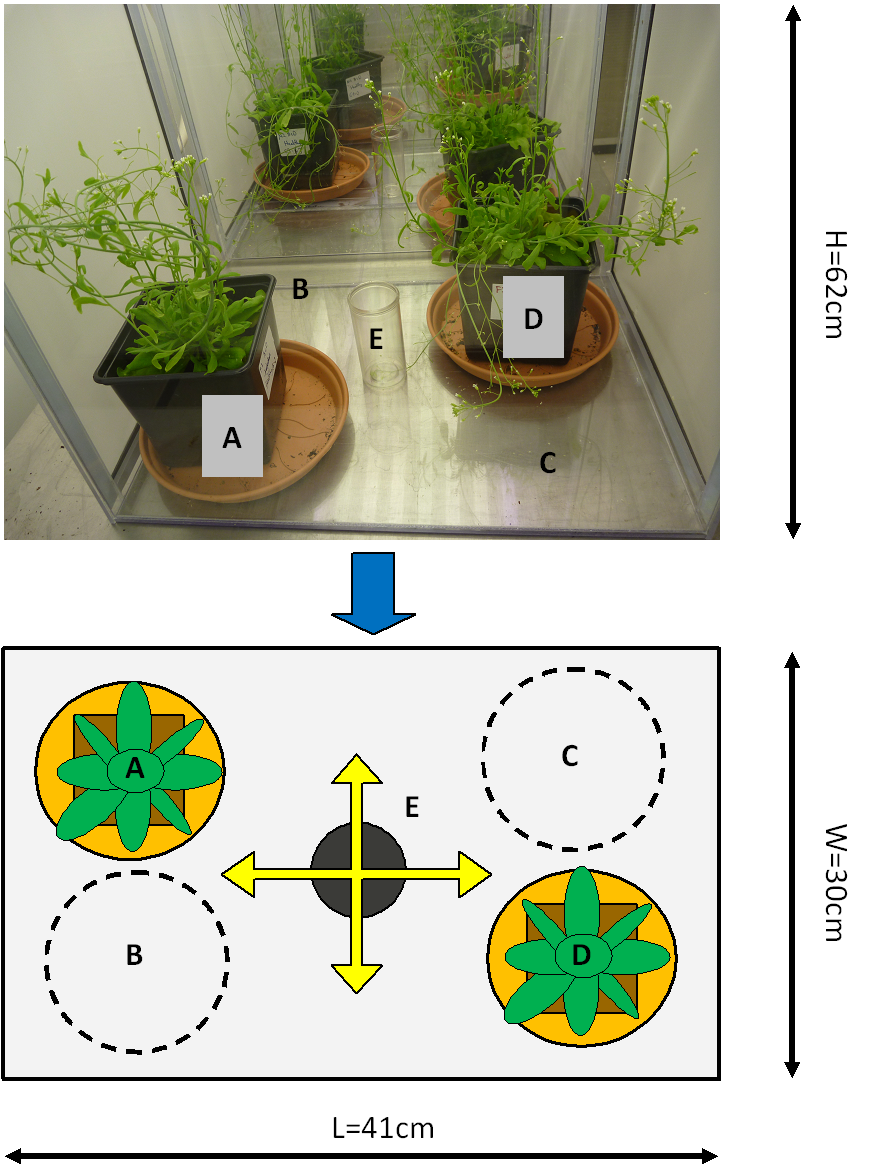

Supplement: Figure S7 — Experimental set-up for insect oviposition choice experiments. Photograph (top) illustrates the actual arrangement of the test plants (A, D) in a choice cage. Several other choice cages are visible in the background with alternative positioning of the test plants. Diagramme (bottom) depicts all available positions for the test plants in the cage (A, B, C, D). Only two positions are occupied in any given cage, resulting from randomly placing the test plants in two out of the four available corners. Insects are introduced in the center of the cage (equidistant from both plants) and released from a transparent plastic tube (E). Arrows indicate the physical dimensions of the cage. (TIFF) [file pbio.1001835.s007.tiff]

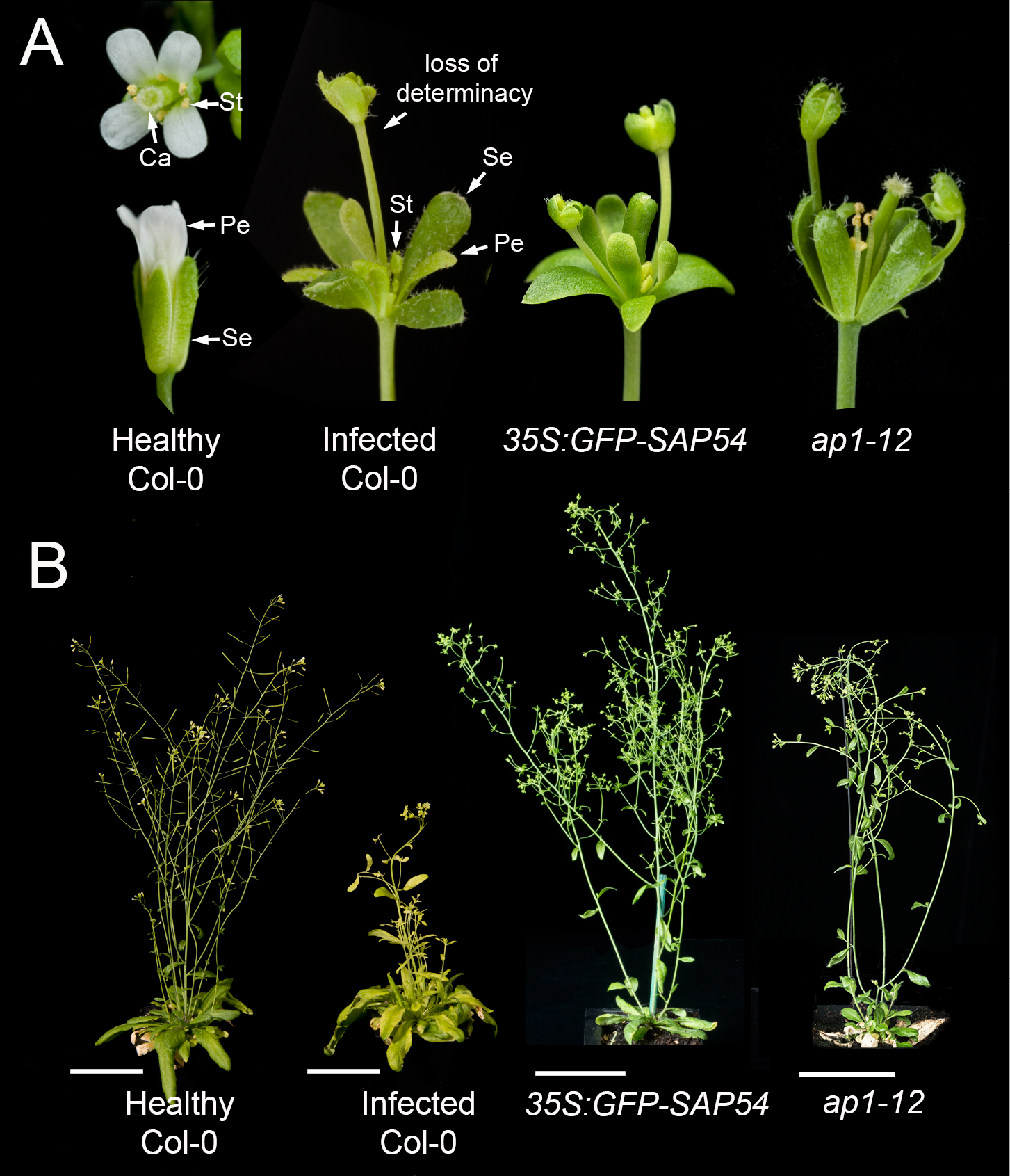

Supplement: Figure S8 — Arabidopsis ap1-12 mutants produce green leaf-like flowers that lack petals. (A) Images of flowers from healthy and AY-WB–infected Arabidopsis wild-type (Col-0) are compared to a GFP–SAP54–expressing transgenic line and ap1-12 mutant. (B) Images of plants representative of healthy and AY-WB–infected Arabidopsis, GFP–SAP54–expressing transgenic lines, and ap1-12 mutants. Scale bars, 5 cm. Se, sepal; Pe, petal; St, stamen; Ca, carpel. (TIFF) [file pbio.1001835.s008.tiff]

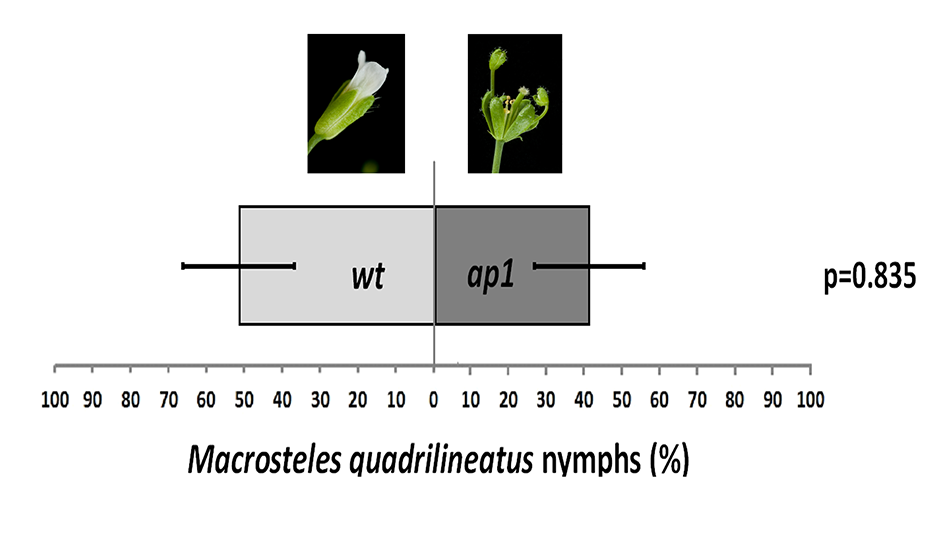

Supplement: Figure S9 — Aster leafhopper Macrosteles quadrilineatus produces a similar number of nymphs on wild-type (wt) and ap1 mutant Arabidopsis plants ( t (11) = 0.22; p = 0.835). (TIFF) [file pbio.1001835.s009.tiff]

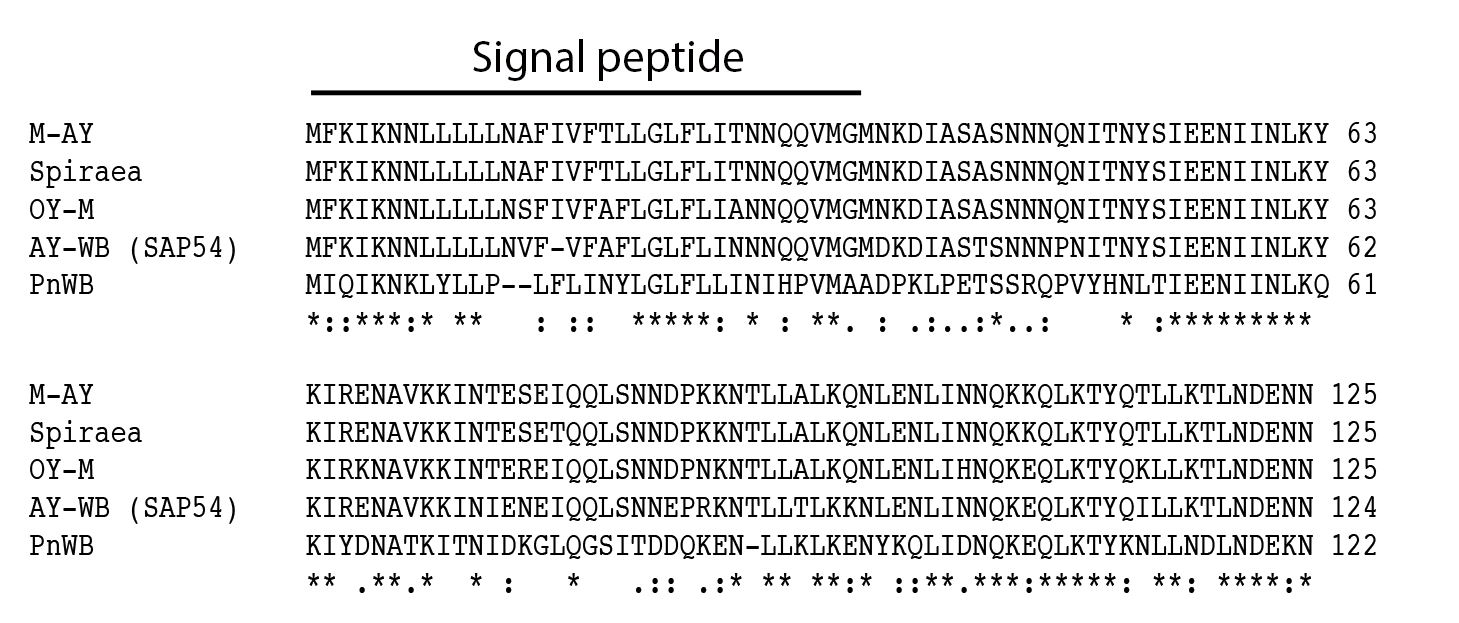

Supplement: Figures S10 — Alignment of amino acid sequences of SAP54 homologues identified in other phytoplasma strains. M-AY, Maryland aster yellows phytoplasma (ABH11652); Spiraea, Spiraea stunt phytoplasma (ABU55747); OY-M, Onion yellows phytoplasma strain OY-M (PAM_049); PnWB, Peanut Witches' Broom phytoplasma (ZP_23918844). (TIFF) [file pbio.1001835.s010.tiff]

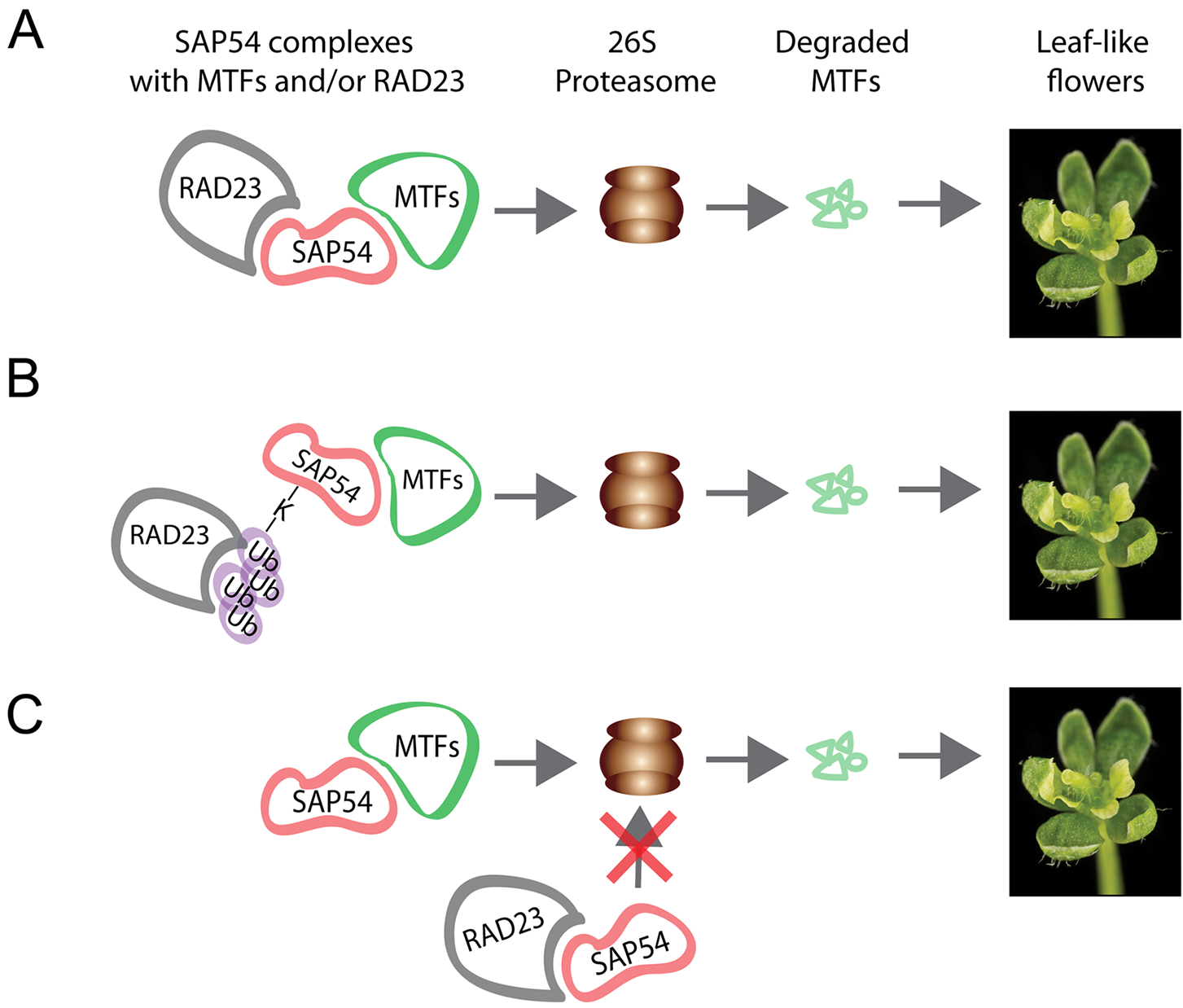

Supplement: Figure S11 — Models of SAP54-mediated degradation of MTFs. (A) SAP54 binds directly to both MTFs and RAD23. The latter takes the SAP54–MTF complex to the plant UPS where the MTFs are degraded. SAP54 may remain associated with RAD23 to prevent being degraded. (B) RAD23 and SAP54 do not interact directly, but via one or more ubiquitin moieties linked via lysine (K) residue(s) on SAP54. RAD23 takes the SAP54–MTF complex to the plant UPS (as in A). (C) An unknown pathway is involved in transportation of SAP54–MTF complexes to the host UPS, whereupon SAP54 interacts with RAD23 to evade degradation. RAD23 and SAP54 may interact directly (as in A) or via ubiquitin (as in B). (TIFF) [file pbio.1001835.s011.tiff]

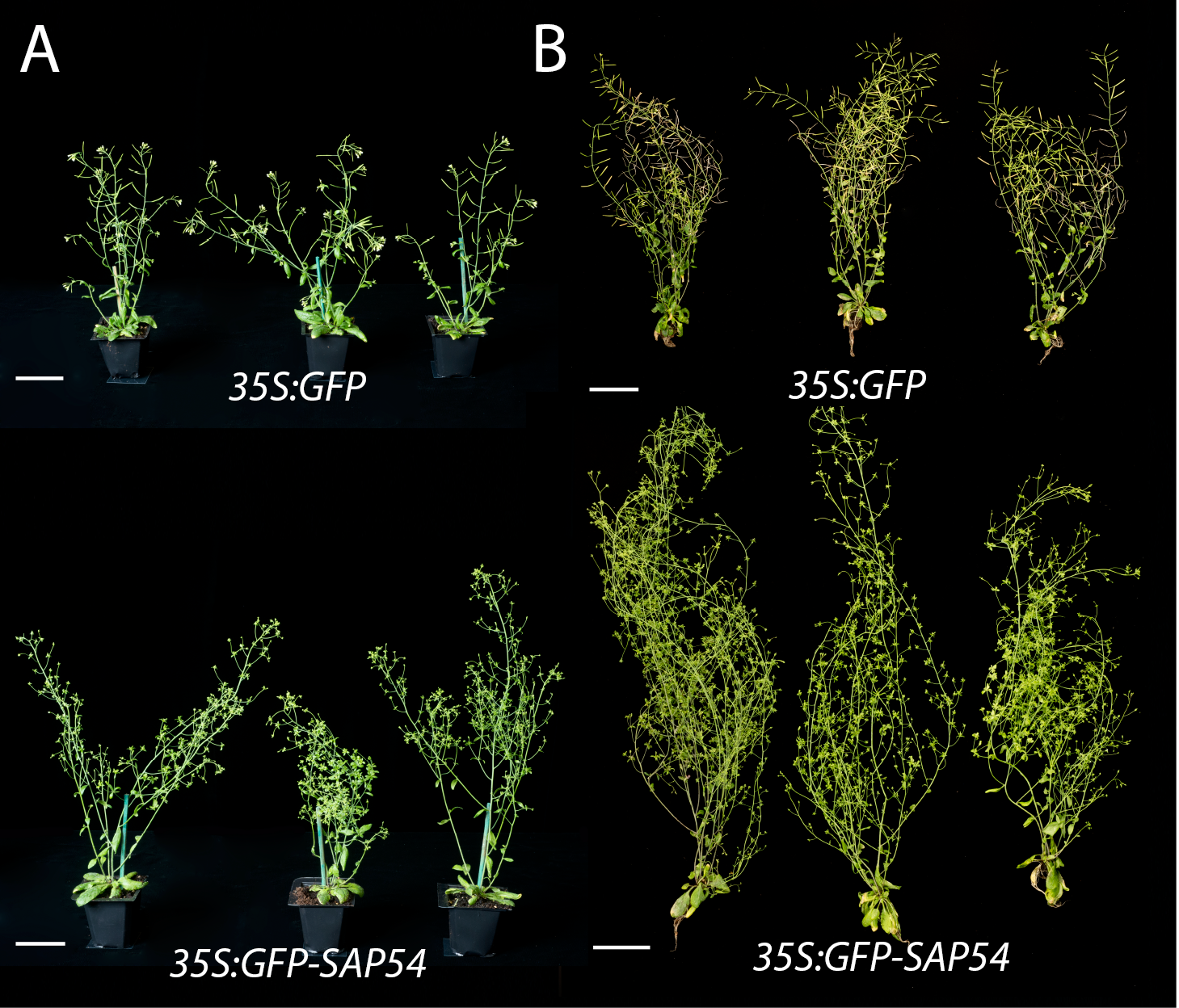

Supplement: Figure S12 — Phytoplasma effector SAP54 alters host development to promote vegetative growth. Shown are 7-wk-old (A) and 10-wk-old (B) transgenic Arabidopsis lines expressing 35S:GFP (control) and 35S:GFP–SAP54. Scale bars, 5 cm. (TIFF) [file pbio.1001835.s012.tiff]
