# Supplementary material for: Phytoplasma Effector SAP54 Hijacks Plant Reproduction by Degrading MADS-box Proteins and Promotes Insect Colonization in a RAD23-Dependent Manner
Source: PLoS Biol. 2014 Apr 8;12(4):e1001835. doi: 10.1371/journal.pbio.1001835 (PMC3979655; doi:10.1371/journal.pbio.1001835)
Supplement: Table S1 — Clones identified as SAP54 interactors in Hybrigenics screen. (DOC) [file pbio.1001835.s013.doc]

**Table S1.** Clones identified as SAP54 interactors in Hybrigenics† screen.

| Clone Name | Genbank ID | Gene name | Interacting sequence (amino acids) |
| --- | --- | --- | --- |
| ATH_RP2_hgx2718v1_pB27_A-247 | 145361954 | SEP3 | 1 - 295 |
| ATH_RP2_hgx2718v1_pB27_B-121 | 145361954 | SEP3 | 1 - 295 |
| ATH_RP2_hgx2718v1_pB27_A-115 | 145361954 | SEP3 | 1 - 295 |
| ATH_RP2_hgx2718v1_pB27_A-109 | 145361954 | SEP3 | 1 - 194 |
| ATH_RP2_hgx2718v1_pB27_A-174 | 145361954 | SEP3 | 1 - 295 |
| ATH_RP2_hgx2718v1_pB27_A-286 | 145361954 | SEP3 | 1 - 295 |
| ATH_RP2_hgx2718v1_pB27_A-143 | 145361954 | SEP3 | 1 - 197 |
| ATH_RP2_hgx2718v1_pB27_A-211 | 145361954 | SEP3 | 1 - 197 |
| ATH_RP2_hgx2718v1_pB27_A-120 | 145361954 | SEP3 | 1 - 197 |
| ATH_RP2_hgx2718v1_pB27_A-150 | 145361954 | SEP3 | 1 - 295 |
| ATH_RP2_hgx2718v1_pB27_A-148 | 145361954 | SEP3 | 1 - 299 |
| ATH_RP2_hgx2718v1_pB27_A-79 | 145361954 | SEP3 | 1 - 240 |
| ATH_RP2_hgx2718v1_pB27_B-112 | 145361954 | SEP3 | 1 - 240 |
| ATH_RP2_hgx2718v1_pB27_A-46 | 145361954 | SEP3 | 1 - 299 |
| ATH_RP2_hgx2718v1_pB27_B-26 | 145361954 | SEP3 | 3 - 223 |
| ATH_RP2_hgx2718v1_pB27_A-85 | 145361954 | SEP3 | 3 - 223 |
| ATH_RP2_hgx2718v1_pB27_A-136 | 145361954 | SEP3 | 3 - 223 |
| ATH_RP2_hgx2718v1_pB27_A-322 | 145361954 | SEP3 | 48 - 214 |
| ATH_RP2_hgx2718v1_pB27_B-8 | 145361954 | SEP3 | 146 - 293 |
| ATH_RP2_hgx2718v1_pB27_A-156 | 30698805 | AGL12 | 2 - 295 |
| ATH_RP2_hgx2718v1_pB27_A-212 | 30698805 | AGL12 | 2 - 295 |
| ATH_RP2_hgx2718v1_pB27_A-309 | 145361960 | MAF1 | 12 - 268 |
| ATH_RP2_hgx2718v1_pB27_B-28 | 145361960 | MAF1 | 12 - 268 |
| ATH_RP2_hgx2718v1_pB27_A-231 | 145361960 | MAF1 | 12 - 268 |
| ATH_RP2_hgx2718v1_pB27_A-288 | 145361960 | MAF1 | 12 - 268 |
| ATH_RP2_hgx2718v1_pB27_A-41 | 145361960 | MAF1 | 7 - 193 |
| ATH_RP2_hgx2718v1_pB27_A-193 | 145361960 | MAF1 | 7 - 193 |
| ATH_RP2_hgx2718v1_pB27_A-99 | 145361960 | MAF1 | 7 - 193 |
| ATH_RP2_hgx2718v1_pB27_B-135 | 145361960 | MAF1 | 12 - 207 |
| ATH_RP2_hgx2718v1_pB27_A-31 | 145361960 | MAF1 | 57 - 284 |
| ATH_RP2_hgx2718v1_pB27_B-62 | 145361960 | MAF1 | 57 - 284 |
| ATH_RP2_hgx2718v1_pB27_A-158 | 145338021 | RAD23C | 130 - 497 |
| ATH_RP2_hgx2718v1_pB27_A-287 | 145338021 | RAD23C | 130 - 497 |
| ATH_RP2_hgx2718v1_pB27_A-84 | 145338021 | RAD23C | 130 - 497 |
| ATH_RP2_hgx2718v1_pB27_B-114 | 145338021 | RAD23C | 132 - 488 |
| ATH_RP2_hgx2718v1_pB27_A-47 | 145338021 | RAD23C | 136 - 473 |
| ATH_RP2_hgx2718v1_pB27_A-205 | 145338021 | RAD23C | 225 - ND |
| ATH_RP2_hgx2718v1_pB27_A-88 | 145338021 | RAD23C | 225 - 513 |
| ATH_RP2_hgx2718v1_pB27_B-73 | 145338021 | RAD23C | 306 - 501 |
| ATH_RP2_hgx2718v1_pB27_B-86 | 145338021 | RAD23C | 380 - 530 |
| ATH_RP2_hgx2718v1_pB27_B-105 | 18421760 | RAD23D | 4 - 442 |
| ATH_RP2_hgx2718v1_pB27_B-35 | 18421760 | RAD23D | 5 - 378 |
| ATH_RP2_hgx2718v1_pB27_A-112 | 18421760 | RAD23D | 20 - 447 |
| ATH_RP2_hgx2718v1_pB27_B-30 | 18421760 | RAD23D | 63 - 444 |
| ATH_RP2_hgx2718v1_pB27_B-146 | 18421760 | RAD23D | 139 - 443 |
| ATH_RP2_hgx2718v1_pB27_B-145 | 18421760 | RAD23D | 162 - 393 |

† Proteins listed in this table have been confirmed to interact with SAP54 in an independent yeast two-hybrid system.
