# Supplementary material for: Phytoplasma Effector SAP54 Hijacks Plant Reproduction by Degrading MADS-box Proteins and Promotes Insect Colonization in a RAD23-Dependent Manner
Source: PLoS Biol. 2014 Apr 8;12(4):e1001835. doi: 10.1371/journal.pbio.1001835 (PMC3979655; doi:10.1371/journal.pbio.1001835)
Supplement: Table S2 — Yeast two-hybrid analysis of SAP54 interactions with MTFs. (DOC) [file pbio.1001835.s014.doc]

**Table S2**. Yeast two-hybrid analysis of SAP54 interactions with MTFs.

| Gene Name | Gene Locus | MADS Lineage | Screen 1:  -His + 1mM 3AT  SAP54 BD/AD | Screen 2:  -Adenine  SAP54 BD/AD |
| --- | --- | --- | --- | --- |
| PHE1 | AT1G65330 | Type I | -/- | NT |
| AGL92 | AT1G31640 | Type I | -/- | NT |
| AGL86 | AT1G31630 | Type I | -/- | NT |
| AGL34 | AT5G26580 | Type I | -/- | NT |
| AGL90 | AT5G27960 | Type I | -/- | NT |
| AGL36 | AT5G26650 | Type I | -/- | NT |
| AGL35 | AT5G26630 | Type I | -/- | NT |
| AGL80 | AT5G48670 | Type I | -/- | NT |
| AGL46 | AT2G28700 | Type I | -/- | NT |
| AGL45 | AT3G05860 | Type I | -/- | NT |
| AGL87 | AT1G22590 | Type I | -/- | NT |
| AGL96 | AT5G06500 | Type I | -/- | NT |
| AGL95 | AT2G15660 | Type I | -/- | NT |
| AGL48 | AT2G40210 | Type I | -/- | NT |
| AGL82 | AT5G58890 | Type I | -/- | NT |
| AGL47 | AT5G55690 | Type I | -/- | NT |
| AGL50 | AT1G59810 | Type I | -/- | NT |
| AGL49 | AT1G60040 | Type I | -/- | NT |
| AGL93 | AT5G26950 | Type I | -/- | NT |
| AGL53 | AT5G27070 | Type I | -/- | NT |
| AGL89 | AT5G27580 | Type I | -/- | NT |
| AGL54 | AT5G27090 | Type I | -/- | NT |
| AGL103 | AT3G18650 | Type I | -/- | NT |
| AGL78 | AT5G65330 | Type I | -/- | NT |
| AGL51 | AT4G02235 | Type I | -/- | NT |
| AGL52 | AT4G11250 | Type I | -/- | NT |
| AGL76 | AT5G40120 | Type I | -/- | NT |
| AGL43 | AT5G40220 | Type I | -/- | NT |
| AGL75 | AT5G41200 | Type I | -/- | NT |
| AGL77 | AT5G38740 | Type I | -/- | NT |
| AGL98 | AT5G39810 | Type I | -/- | NT |
| AGL81 | AT5G39750 | Type I | -/- | NT |
| AGL57 | AT3G04100 | Type I | -/- | NT |
| AGL91 | AT3G66656 | Type I | -/- | NT |
| AGL29 | AT2G34440 | Type I | -/- | NT |
| AGL85 | AT1G54760 | Type I | -/- | NT |
| AGL58 | AT1G28450 | Type I | -/- | NT |
| AGL59 | AT1G28460 | Type I | -/- | NT |
| AGL102 | AT1G47760 | Type I | -/- | NT |
| AGL83 | AT5G49490 | Type I | -/- | NT |
| AGL73 | AT5G38620 | Type I | -/- | NT |
| AGL99 | AT5G04640 | Type I | -/- | NT |
| AGL97 | AT1G46408 | Type I | -/- | NT |
| AGL56 | AT1G60880 | Type I | -/- | NT |
| AGL55 | AT1G60920 | Type I | -/- | NT |
| AGL39 | AT5G27130 | Type I | -/- | NT |
| DIANA | AT2G24840 | Type I | -/- | NT |
| AGL62 | AT5G60440 | Type I | -/- | NT |
| AGL40 | AT4G36590 | Type I | -/- | NT |
| AGL23 | AT1G65360 | Type I | -/- | NT |
| AGL28 | AT1G01530 | Type I | -/- | NT |
| AGL65 | AT1G18750 | Type II (MIKC*-P) | -/- | NT |
| AGL30 | AT2G03060 | Type II (MIKC*-P) | -/- | NT |
| AGL94 | AT1G69540 | Type II (MIKC*-P) | -/- | NT |
| AGL66 | AT1G77980 | Type II (MIKC*-S) | -/- | NT |
| AGL104 | AT1G22130 | Type II (MIKC*-S) | -/- | NT |
| PI | AT5G20240 | Type II (MIKC) | -/- | NT |
| AP3 | AT3G54340 | Type II (MIKC) | -/- | NT |
| ABS | AT5G23260 | Type II (MIKC) | -/- | NT |
| GOA | AT1G31140 | Type II (MIKC) | -/- | NT |
| FLC | AT5G10140 | Type II (MIKC) | (+)/- | -/- |
| MAF1/FLM (alpha)^†^ | AT1G77080.5 | Type II (MIKC) | -/+ | -/- |
| MAF1/FLM (beta)^†^ | AT1G77080.4 | Type II (MIKC) | -/+ | -/+ |
| MAF1/FLM (delta)^†^ | AT1G77080.2 | Type II (MIKC) | -/- | -/- |
| MAF2 | AT5G65050 | Type II (MIKC) | -/- | NT |
| MAF3 | AT5G65060 | Type II (MIKC) | -/- | NT |
| MAF4 | AT5G65070 | Type II (MIKC) | -/- | NT |
| MAF5 | AT5G65080 | Type II (MIKC) | -/++ | -/- |
| SEP1 | AT5G15800 | Type II (MIKC) | ++/AA | ++/- |
| SEP2 | AT3G02310 | Type II (MIKC) | -/++ | -/++ |
| SEP3 | AT1G24260 | Type II (MIKC) | ++/++ | ++/++ |
| SEP4 (I)^†^ | AT2G03710 | Type II (MIKC) | ++/- | ++/- |
| SEP4 (II)^†^ | AT2G03710 | Type II (MIKC) | +/++ | +/++ |
| AGL13 | AT3G61120 | Type II (MIKC) | -/- | NT |
| AGL6 | AT2G45650 | Type II (MIKC) | ++/AA | ++/- |
| FUL | AT5G60910 | Type II (MIKC) | (+)/- | -/- |
| AP1 | AT1G69120 | Type II (MIKC) | ++/AA(++) | ++/++ |
| CAL | AT1G26310 | Type II (MIKC) | (+)/- | -/- |
| AGL79 | AT3G30260 | Type II (MIKC) | -/+ | -/+ |
| AGL12/XAN | AT1G71692 | Type II (MIKC) | -/+ | -/+ |
| AGL14 | AT4G11880 | Type II (MIKC) | -/+ | -/- |
| AGL19 | AT4G22950 | Type II (MIKC) | -/- | NT |
| SOC1 | AT2G45660 | Type II (MIKC) | ++/++ | -/- |
| AGL71 | AT5G51870 | Type II (MIKC) | -/- | NT |
| AGL72 | AT5G51860 | Type II (MIKC) | -/- | NT |
| AGL42 | AT5G62165 | Type II (MIKC) | -/- | NT |
| AGL16 | AT3G57230 | Type II (MIKC) | -/- | NT |
| AGL17 | AT2G22630 | Type II (MIKC) | -/- | NT |
| AGL21 | AT4G37940 | Type II (MIKC) | -/- | NT |
| AGL44/ANR1 | AT2G14210 | Type II (MIKC) | -/- | NT |
| AGL15 | AT5G13790 | Type II (MIKC) | -/- | NT |
| AGL18 | AT3G57390 | Type II (MIKC) | -/- | NT |
| AGL24 | AT4G24540 | Type II (MIKC) | -/- | NT |
| SVP1/AGL22 | AT2G22540 | Type II (MIKC) | -/- | NT |
| AG | AT4G18960 | Type II (MIKC) | -/- | NT |
| STK | AT4G09960 | Type II (MIKC) | -/- | NT |
| SHP1 | AT3G58780 | Type II (MIKC) | -/- | NT |
| SHP2 | AT2G42830 | Type II (MIKC) | -/- | NT |

† Details regarding splice variants of these proteins have been described in Severing et al., (*28*)

NT: not tested; AA: not determined due to autoactivation; - : no growth; (+): very weak growth; + : intermediate growth; ++ : strong growth; AA(++): increased growth above the autoactivation level for this bait, indicating an interaction event.
