# Supplementary material for: Phytoplasma Effector SAP54 Hijacks Plant Reproduction by Degrading MADS-box Proteins and Promotes Insect Colonization in a RAD23-Dependent Manner
Source: PLoS Biol. 2014 Apr 8;12(4):e1001835. doi: 10.1371/journal.pbio.1001835 (PMC3979655; doi:10.1371/journal.pbio.1001835)
Supplement: Table S3 — Quantification of signal intensity levels (ImageJ) of bands in Figure 1D . (DOC) [file pbio.1001835.s015.doc]

**Table S3.** Quantification of signal intensity levels (ImageJ) of bands in Fig. 1D.

| **Lane** | **IP** | | **Input** | | **Input protein concentration (mg/ml)**^†^ | **IP α-SAP54 signal intensity**  **/input protein concentration** |
| --- | --- | --- | --- | --- | --- | --- |
|  | **α-GFP** | **α-SAP54** | **α-GFP** | **α-SAP54** |  |  |
| SEP3-GFP healthy | 9142.51 | 167.36 | 6018.80 | 0.00 | 3.60 | 46.48 |
| SEP3-GFP infected | 14734.44 | 10362.46 | 8070.53 | 5889.07 | 4.72 | 2195.44* |
| FUL-GFP healthy | 10424.48 | 0.00 | 8055.92 | 0.00 | 2.02 | 0.00 |
| FUL-GFP infected | 18791.40 | 0.00 | 3796.83 | 5579.00 | 4.02 | 0.00 |
| AG-GFP healthy | 10172.24 | 257.43 | 6614.46 | 0.00 | 3.01 | 85.52 |
| AG-GFP infected | 14312.16 | 0.00 | 4179.25 | 8714.32 | 3.60 | 0.00 |

^†^ Determined via Bradford assay.

*Higher value suggests SAP54 pull down with SEP3-GFP.
