# Supplementary material for: Phytoplasma Effector SAP54 Hijacks Plant Reproduction by Degrading MADS-box Proteins and Promotes Insect Colonization in a RAD23-Dependent Manner
Source: PLoS Biol. 2014 Apr 8;12(4):e1001835. doi: 10.1371/journal.pbio.1001835 (PMC3979655; doi:10.1371/journal.pbio.1001835)
Supplement: Table S4 — Mass spectrometry analysis of MTFs that interact with GFP–SAP54. (DOC) [file pbio.1001835.s016.doc]

**Table S4.** Mass spectrometry analysis of MTFs that interact with GFP-SAP54.

| **Protein** | **Gene locus** | **Number of unique peptides** | **Peptide sequence** | **Percent**  **coverage of protein sequence** |
| --- | --- | --- | --- | --- |
| SOC1 | AT2G45660 | 7 | LYEFASSNMQDTIDR  VSTKPVSEENMQHLK  IEQLEASK  KIEQLEASK  KIEQLEASKR  LLGEGIGTCSIEELQQIEQQLEK  WGSHESEVWSNK | 35 |
| MAF1 | AT1G77080 | 3 | IIDRYEIQHADELR  EENQVLASQMGK  NTLLATDDER | 18 |
| AP1 | AT1G69120 | 1 | AIQEQNSMLSK | 4 |
| SEP2 | AT3G02310 | 1 | EHILLDANR | 4 |
| SEP1 | AT5G15800 | 1 | TQYMLDQLSDLQNK | 6 |
