# Supplementary material for: Phytoplasma Effector SAP54 Hijacks Plant Reproduction by Degrading MADS-box Proteins and Promotes Insect Colonization in a RAD23-Dependent Manner
Source: PLoS Biol. 2014 Apr 8;12(4):e1001835. doi: 10.1371/journal.pbio.1001835 (PMC3979655; doi:10.1371/journal.pbio.1001835)
Supplement: Table S5 — Signal intensity levels (ImageJ) of bands in Figure 2A . (DOC) [file pbio.1001835.s017.doc]

**Table S5. Signal intensity levels (ImageJ) of bands in Fig. 2A.**

| **Lane** | **α-GFP** | **α-SAP54** | **Loading** | **Ratio α-GFP/loading** |
| --- | --- | --- | --- | --- |
| AP1-GFP healthy | 22224.47 | 2410.91 | 5726.78 | 3.88 |
| AP1-GFP infected | 15520.52 | 8919.60 | 8563.73 | 1.81* |
| SEP3-GFP healthy | 7870.63 | 429.26 | 6467.49 | 1.21 |
| SEP3-GFP infected | 1943.91 | 5791.88 | 8267.25 | 0.24* |

*Lower values in this row compared to the value in the row above indicate degradation of AP1 or SEP3
