# Supplementary material for: Phytoplasma Effector SAP54 Hijacks Plant Reproduction by Degrading MADS-box Proteins and Promotes Insect Colonization in a RAD23-Dependent Manner
Source: PLoS Biol. 2014 Apr 8;12(4):e1001835. doi: 10.1371/journal.pbio.1001835 (PMC3979655; doi:10.1371/journal.pbio.1001835)
Supplement: Table S6 — Signal intensity levels (ImageJ) of bands in Figure 2B . (DOC) [file pbio.1001835.s018.doc]

**Table S6. Signal intensity levels (ImageJ) of bands in Fig. 2B.**

| **Lane** | **α-myc** | **α-flag** | **α-SAP54** | **Loading** | **Ratio**  **α-myc/loading** |
| --- | --- | --- | --- | --- | --- |
| 10xmyc-AP1 x flag-RFP | 17877.64 | 66837.19 | 0.00 | 9209.20 | 1.94 |
| 10xmyc-AP1 x flag-SAP54 | 894.02 | 53657.04 | 23376.02 | 8684.85 | 0.10* |
| flag-RFP (no myc-tagged MTF) | 0.00 | 33204.72 | 6914.37 | 9811.44 | 0.00 |
| 10xmyc-SEP3 x flag-RFP | 18549.16 | 58296.18 | 182.02 | 15115.68 | 1.23 |
| 10xmyc-SEP3 x flag-SAP54 | 614.80 | 15190.08 | 11550.39 | 11168.25 | 0.06* |
| 10xmyc-SOC1 x flag-RFP | 33936.60 | 68532.23 | 0.00 | 8868.32 | 3.83 |
| 10xmyc-SOC1 x flag-SAP54 | 9188.72 | 30075.02 | 7454.88 | 8222.02 | 1.12* |

*Lower values in this row compared to the value in the row above indicate degradation of AP1, SEP3 or SOC1
