# Supplementary material for: Phytoplasma Effector SAP54 Hijacks Plant Reproduction by Degrading MADS-box Proteins and Promotes Insect Colonization in a RAD23-Dependent Manner
Source: PLoS Biol. 2014 Apr 8;12(4):e1001835. doi: 10.1371/journal.pbio.1001835 (PMC3979655; doi:10.1371/journal.pbio.1001835)
Supplement: Table S7 — Signal intensity levels (ImageJ) of bands in Figure 2C . (DOC) [file pbio.1001835.s019.doc]

**Table S7.** Signal intensity levels (ImageJ) of bands in Fig. 2C.

| **Lane** | **Treatment** | **α-myc** | **α-flag** | **α-SAP54** | **Loading** | **Ratio αmyc/ loading** |
| --- | --- | --- | --- | --- | --- | --- |
| 10xmyc-AP1 x flag-RFP | none | 9162.25 | 29335.87 | 0.00 | 8425.20 | 1.09 |
| 10xmyc-AP1 x flag-SAP54 | none | 2932.57 | 7666.83 | 9662.71 | 9624.63 | 0.30* |
| 10xmyc-AP1 x flag-SAP54 | DMSO | 5310.52 | 9890.20 | 9390.22 | 8202.78 | 0.65 |
| 10xmyc-AP1 x flag-SAP54 | Epoxomicin | 53016.47 | 8697.83 | 8525.35 | 7765.02 | 6.83** |

*Lower value in this row compared to the value in the row above indicates degradation of AP1; **Higher value in this row compared to the row above indicates inhibition of degradation of AP1.
