# Supplementary material for: Phytoplasma Effector SAP54 Hijacks Plant Reproduction by Degrading MADS-box Proteins and Promotes Insect Colonization in a RAD23-Dependent Manner
Source: PLoS Biol. 2014 Apr 8;12(4):e1001835. doi: 10.1371/journal.pbio.1001835 (PMC3979655; doi:10.1371/journal.pbio.1001835)
Supplement: Table S8 — Signal intensity levels (ImageJ) of IP bands in Figure 2D . (DOC) [file pbio.1001835.s020.doc]

**Table S8.** Signal intensity levels (ImageJ) of IP bands in Fig. 2D.

| **Lane (Treatment)** | **IP** | | **Ratio α-myc/**  **α-GFP** |
| --- | --- | --- | --- |
|  | **α-myc** | **α-GFP** |  |
| 10xmyc-AP1 x GFP (DMSO) | 0.00 | 14404.39 | 0.00 |
| 10xmyc-AP1 x GFP-SAP54 (DMSO) | 7076.58 | 11794.52 | 0.60 |
| 10xmyc-AP1 x GFP-SAP54 (Epoxomicin) | 24961.10 | 11014.84 | 2.27* |
| 10xmyc-SEP3 x GFP/DMSO | 0.00 | 13570.53 | 0.00 |
| 10xmyc-SEP3 x GFP-SAP54 (DMSO) | 9959.33 | 10535.97 | 0.95 |
| 10xmyc-SEP3 x GFP-SAP54 (Epoxomicin) | 22046.32 | 15332.65 | 1.44* |
| 10xmyc-SOC1 x GFP (DMSO) | 0.00 | 10696.22 | 0.00 |
| 10xmyc-SOC1 x GFP-SAP54 (DMSO) | 21476.81 | 6001.30 | 3.58* |
| 10xmyc-SOC1 x GFP (Epoxomicin) | 0.00 | 9451.82 | 0.00 |
| 10xmyc-SOC1 x GFP-SAP54 (Epoxomicin) | 17042.04 | 3943.98 | 4.32* |
| 10xmyc-AGL50 x GFP (DMSO) | 0.00 | 9175.15 | 0.00 |
| 10xmyc-AGL50 x GFP-SAP54 (DMSO) | 0.00 | 5384.30 | 0.00 |
| 10xmyc-AGL50 x GFP-SAP54 (Epoxomicin) | 0.00 | 5796.22 | 0.00 |

*Higher numbers compared to the rows above indicate pull down of AP1, SEP3 or SOC1.
