# Supplementary material for: Phytoplasma Effector SAP54 Hijacks Plant Reproduction by Degrading MADS-box Proteins and Promotes Insect Colonization in a RAD23-Dependent Manner
Source: PLoS Biol. 2014 Apr 8;12(4):e1001835. doi: 10.1371/journal.pbio.1001835 (PMC3979655; doi:10.1371/journal.pbio.1001835)
Supplement: Table S9 — Signal intensity levels (ImageJ) of input bands in Figure 2D . (DOC) [file pbio.1001835.s021.doc]

**Table S9. Signal intensity levels (ImageJ) of input bands in Fig. 2D.**

| **Lane (Treatment)** | **Input** | | | | **Ratio**  **α-myc/loading** |
| --- | --- | --- | --- | --- | --- |
|  | **α-GFP** | **α-SAP54** | **α-myc** | **Loading** |  |
| 10xmyc-AP1 x GFP (DMSO) | 21806.74 | 0.00 | 4047.05 | 6161.64 | 0.66 |
| 10xmyc-AP1 x GFP-SAP54 (DMSO) | 18805.95 | 13606.46 | 2479.93 | 6952.54 | 0.36 |
| 10xmyc-AP1 x GFP-SAP54 (Epoxomicin) | 19942.71 | 16661.77 | 12434.48 | 6948.23 | 1.79 |
| 10xmyc-SEP3 x GFP/DMSO | 22564.09 | 0.00 | 5789.52 | 10676.23 | 0.54 |
| 10xmyc-SEP3 x GFP-SAP54 (DMSO) | 30940.50 | 19458.19 | 8147.75 | 10315.42 | 0.79 |
| 10xmyc-SEP3 x GFP-SAP54 (Epoxomicin) | 24920.68 | 19595.02 | 9079.24 | 10326.49 | 0.88 |
| 10xmyc-SOC1 x GFP (DMSO) | 24320.07 | ND* | ND* | 11796.13 | ND* |
| 10xmyc-SOC1 x GFP-SAP54 (DMSO) | 8184.54 | ND* | ND* | 10812.17 | ND* |
| 10xmyc-SOC1 x GFP (Epoxomicin) | 23921.50 | ND* | ND* | 11459.54 | ND* |
| 10xmyc-SOC1 x GFP-SAP54 (Epoxomicin) | 11953.87 | ND* | ND* | 11136.59 | ND* |
| 10xmyc-AGL50 x GFP (DMSO) | 26541.41 | ND* | 13811.44 | 11405.00 | 1.21 |
| 10xmyc-AGL50 x GFP-SAP54 (DMSO) | 21402.93 | ND* | 13831.15 | 10192.18 | 1.36 |
| 10xmyc-AGL50 x GFP-SAP54 (Epoxomicin) | 13937.15 | ND* | 12116.90 | 10195.88 | 1.89 |

*Not determined.
