# Supplementary material for: Phytoplasma Effector SAP54 Hijacks Plant Reproduction by Degrading MADS-box Proteins and Promotes Insect Colonization in a RAD23-Dependent Manner
Source: PLoS Biol. 2014 Apr 8;12(4):e1001835. doi: 10.1371/journal.pbio.1001835 (PMC3979655; doi:10.1371/journal.pbio.1001835)
Supplement: Table S10 — List of primers used in study. (DOC) [file pbio.1001835.s022.doc]

**Table S10.** List of primers used in study.

| Primer name | Sequence (5' - 3') | Comment |
| --- | --- | --- |
| attB1 adaptor | GGGGACAAGTTTGTACAAAAAAGCAGGC | used with attB2 adaptor primer to reconstitute attB sites for BP clonase rxn |
| attB2 adaptor | GGG GAC CAC TTT GTA CAA GAA AGC TGG GT | used with attB1 adaptor primer to reconstitute attB sites for BP clonase rxn |
| attB1AGL50 | AAAAAGCAGGCTccaccatgGCTCCTCGTCAGAAGAAACCTAAC | to clone AGL50 into pDONR207 |
| attB2AGL50 | AGAAAGCTGGGTgTTAAATTGAAATCATATCGGTGCTCATCG | to clone AGL50 into pDONR207 |
| attB1AGL62 | AAAAAGCAGGCTccaccatgGTGAAAAAAAGCAAAGGTCGTC | to clone AGL62 into pDONR207 |
| attB2AGL62 | AGAAAGCTGGGTgTTAATAGTAATCAGATCTAGACTGGG | to clone AGL62 into pDONR207 |
| attB1AGL80 | AAAAAGCAGGCTccaccatgACAAGAAAGAAAGTGAAACTTGC | to clone AGL80 into pDONR207 |
| attB2AGL80 | AGAAAGCTGGGTgTTAATGGAACCATGTTTTATTGGTAATAC | to clone AGL80 into pDONR207 |
| attB1AGL66 | AAAAAGCAGGCTccaccatgGGTCGAGTGAAATTGGAGATAAAACG | to clone AGL66 into pDONR207 |
| attB2AGL66 | AGAAAGCTGGGTgTTATTGGCTACTGAGCTGAGGC | to clone AGL66 into pDONR207 |
| attB1SEP3 | AAAAAGCAGGCTccaccatgGGAAGAGGGAGAGTAGAATTGAAGAGG | to clone SEP3 into pDONR207 |
| attB2SEP3 | AGAAAGCTGGGTgTCAAATAGAGTTGGTGTCATAAGGTAACC | to clone SEP3 into pDONR207 |
| attB1CAL | AAAAAGCAGGCTccaccatgGGAAGGGGTAGGGTTGAATTGAAGAGG | to clone CAL into pDONR207 |
| attB2CAL | AGAAAGCTGGGTgTCAAGCGGCGTAACAGCCAAGGTAATTGTAAATGG | to clone CAL into pDONR207 |
| attB1FUL | AAAAAGCAGGCTccaccatgGGAAGAGGTAGGGTTCAGC | to clone FUL into pDONR207 |
| attB2FUL | AGAAAGCTGGGTgCTACTCGTTCGTAGTGGTAGG | to clone FUL into pDONR207 |
| attB1SOC1 | AAAAAGCAGGCTccaccatgGTGAGGGGCAAAACTCAGATGAAG | to clone SOC1 into pDONR207 |
| attB2SOC1 | AGAAAGCTGGGTgTCACTTTCTTGAAGAACAAGGTAACC | to clone SOC1 into pDONR207 |
| attB1AP1 | AAAAAGCAGGCTccaccatgGGAAGGGGTAGGGTTCAATT | to clone AP1 into pDONR207 |
| attB2AP1 | AGAAAGCTGGGTgTCATGCGGCGAAGCAGCCAA | to clone AP1 into pDONR207 |
| attB1RAD23A | AAAAAGCAGGCTccaccatgAAGCTCACTGTTAAGACTCTCAAGGGTAGC | to clone RAD23A into pDONR207 |
| attB2RAD23A | AGAAAGCTGGGTgTCAGTCTTCAAAATCTGCTGAGTGCTCTAG | to clone RAD23A into pDONR207 |
| attB1RAD23B | AAAAAGCAGGCTccaccatgAAGCTCACCGTTAAAACTCTCAAGGGCAGT | to clone RAD23B into pDONR207 |
| attB2RAD23B | AGAAAGCTGGGTgTCAGTCTTCAAAGTCACCTGAGTTCTCC | to clone RAD23B into pDONR207 |
| attB1RAD23C | AAAAAGCAGGCTccaccatgAAGATATTTGTGAAAACTCTCAAGGGGACT | to clone RAD23C into pDONR207 |
| attB2RAD23C | AGAAAGCTGGGTgTTATTCCTCGAATTCATGCATGTGATCTAGAAG | to clone RAD23C into pDONR207 |
| attB1RAD23D | AAAAAGCAGGCTccaccatgAAGATTTTCGTGAAGACTCTCAGTGGTTCG | to clone RAD23D into pDONR207 |
| attB2RAD23D | AGAAAGCTGGGTgTTATTGATCTTCAAACTCATGCATGTGATC | to clone RAD23D into pDONR207 |
| AP1MADsRev | AGAAAGCTGGGTgTCAGACGTCGGACTCAGGTGCAATAAGC | used with attB1AP1 to clone MADS + Intervening domains of AP1 into pDONR207 |
| AP1KdomFor | AAAAAGCAGGCTccaccatgAATACAAACTGGTCGATGGAGTATAAC | used with AP1KdomRev to clone Keratin-like domain of AP1 into pDONR207 |
| AP1KdomRev | AGAAAGCTGGGTgTCAAATTTTTTCCCTCTCCTTGATCTGTTTAG | used with AP1KdomFor to clone Keratin-like domain of AP1 into pDONR207 |
| AP1CtermFor | AAAAAGCAGGCTccaccatgCTTAGGGCTCAACAGGAGCAGTGG | used with attB2AP1 to clone C-terminal domain of AP1 into pDONR207 |
| pTRBOSAP54F | atacctaggATGGACTACAAGGACGACGATGACAAAATGGATAAAGATAT-  TGCTAGC | used to clone Flag-SAP54 into pTRBO via AvrII and NotI sites |
| pTRBOSAP54R | ATAGCGGCCGCTTAATTATTTTCATCATTTAAAGTT | used to clone Flag-SAP54 into pTRBO via AvrII and NotI sites |
| attB1SAP54F | AAAAAGCAGGCTccaccATGGATAAAGATATTGCTAGC | used to clone SAP54 into pDONR207 |
| attB2SAP54R | AGAAAGCTGGGTgTTAATTATTTTCATCATTTAAAG | used to clone SAP54 into pDONR207 |
| attB1foreGFP | AAAAAGCAGGCTccaccatgGTGAGCAAGGGCGAGGAGCTG | used to clone eGFP into pDONR207 |
| attB2reveGFP | AGAAAGCTGGGTgTTACTTGTACAGCTCGTCCATGCCGAG | used to clone eGFP  into pDONR207 |
| EF-1For | CTGCTGTTGTAACAAGATGGATGCCA | Arabidopsis elongation  factor 1-alpha |
| EF-1Rev | TGCAGCCTTGGTAACCTTGGCTCC | Arabidopsis elongation  factor 1-alpha |
| BF | AGGATGGAACCCTTCAATGTC | AY-WB specific primers [30] |
| BR | GGAAGTCGCCTACAAAAATCC | AY-WB specific primers [30] |
